# N-glycosylation of PD-L1 modulates the efficacy of immune checkpoint blockades targeting PD-L1 and PD-1

**DOI:** 10.1186/s12943-025-02330-w

**Published:** 2025-05-10

**Authors:** Bar Kaufman, Muhammad Abu-Ahmad, Olga Radinsky, Eman Gharra, Tal Manko, Baisali Bhattacharya, Daniela Gologan, Nofar Erlichman, Tsipi Meshel, Yoav Nuta, Tomer Cooks, Moshe Elkabets, Adit Ben-Baruch, Angel Porgador

**Affiliations:** 1 The Shraga Segal Department of Microbiology, Immunology and Genetics, Faculty of Health Sciences, Ben-Gurion University of the Negev, Beer-Sheva, 84105 Israel; 2 The Shmunis School of Biomedicine and Cancer Research, George S. Wise Faculty of Life Sciences, Tel Aviv University, Tel Aviv, 6997801 Israel

**Keywords:** PD-L1, PD-1, N-glycosylation, Inhibitory immune checkpoints, T-cell activation, Cancer immunotherapy, Anti-PD-L1/PD-1 antibodies, Glycosylation mutants, Degranulation assay, Cytotoxicity

## Abstract

**Background:**

The PD-L1/PD-1 pathway is crucial for immune regulation and has become a target in cancer immunotherapy. However, in order to improve patient selection for immune checkpoint blockade (ICB) therapies, better selection criteria are needed. This study explores how the N-glycosylation of PD-L1 affects its interaction with PD-1 and ICB efficacy, focusing on its four N-linked glycosylation sites: N35, N192, N200, and N219.

**Methods:**

Human PD-L1 glycosylation mutants—at each individual site or at all four sites together (Nx4)—were tested for their functional interaction with PD-1 using an artificial immune checkpoint reporter assay (IcAR-PD1). The blocking efficacy of anti-PD-L1 and anti-PD-1 antibodies was evaluated using human breast cancer cell lines (MDA-MB231 and MCF7), as well as A375 melanoma and A549 lung carcinoma cells expressing the glycosylation mutants. Results were validated through ex vivo activation and cytotoxicity assays using human CD8+ T cells.

**Results:**

The binding of the PD-L1_N35A_ mutant to PD-1 was not effectively blocked by anti-PD-L1 and anti-PD-1 ICBs. In contrast, high blocking efficacy of PD-L1 binding to PD-1 was obtained at minimal ICB concentrations when PD-L1 did not express any glycosylation site (PD-L1_Nx4_ mutant). The PD-L1_N35A_ mutant produced elevated levels of PD-L1 as a soluble (sPD-L1) and extracellular vesicles (EV)-bound molecule; in contrast, the PD-L1_Nx4_ mutant had lower sPD-L1 and EV levels. PD-L1 glycosylation status influenced the ability of PD-L1 interactions with PD-1 to down-regulate T-cell activation and cytotoxicity, with the PD-L1_N35A_ mutant showing the lowest levels of T cell functions and the PD-L1_Nx4_ mutant the highest.

**Conclusions:**

The N-glycosylation of PD-L1 at all four sites interferes with the ability of anti-PD-L1 and anti-PD-1 ICBs to block PD-L1 interactions with PD-1; in contrast, glycosylation at the N35 site enhances ICB blocking efficacy. These effects are connected to the ability of sPD-L1 to compete with ICB binding to PD-L1 or PD-1. Thus, assessing PD-L1 glycosylation, beyond expression levels, could improve patient stratification and outcomes.

**Supplementary Information:**

The online version contains supplementary material available at 10.1186/s12943-025-02330-w.

## Background

The programmed death-ligand 1 (PD-L1) and programmed cell death protein 1 (PD-1) pathway has emerged as a pivotal mechanism in regulating immune responses, including in cancer [1, 2]. PD-L1 is often up-regulated in tumor cells [3] whereas PD-1 is expressed by activated T-cells [4]. Interactions between PD-L1 and PD-1 often lead to immune suppression and T-cell exhaustion, contributing to cancer cell evasion from immune surveillance [5]. Moreover, PD-L1 has major non-immune functions that promote tumor progression, including cell-autonomous and PD-1-induced pro-metastatic functions in cancer cells [6, 7], elevated expression of PD-L1 by cancer stem cells, and epithelial-to-mesenchymal differentiation regulation [8].

The interactions between PD-L1 and PD-1 have become a focal point in cancer immunotherapy [9, 10], and immune checkpoint blockades (ICBs) targeting the PD-L1/PD-1 axis demonstrate significant clinical efficacy in various malignancies [11–13]. However, the response to these therapies is variable and only a subset of patients achieves durable benefits [14]. Moreover, while PD-L1 staining is intended to guide treatment decisions, its effectiveness in predicting patient response to therapy is often limited [14, 15].

These findings underline the need for more understanding of the mechanisms that regulate PD-L1 activities and its interactions with PD-1. Post-translational modifications [16], particularly N-glycosylation, play a significant role in regulating protein function and stability [17]. PD-L1 contains four N-glycosylation sites: N35, N192, N200, and N219 [18]. N-glycosylation of PD-L1 plays key roles in stabilizing the protein [19] and facilitating its localization to cell membranes [20–22], suggesting that this modification may influence PD-L1’s ability to promote immune evasion in cancer. Additionally, surface plasmon resonance (SPR) studies indicate that N-glycosylation of PD-L1 affects how it interacts with anti-PD-L1 antibodies [23].

While the significance of the overall N-glycosylation to PD-L1 regulation and impact on immune activities was reported [24, 25], the specific contributions of individual glycosylation sites to PD-L1 interactions with PD-1 was minimally addressed. Moreover, the effect of N-glycosylation of PD-L1 on the efficacy of ICBs targeting PD-L1 or PD-1, remains inadequately understood. Investigating these mechanisms is essential for enhancing ICB efficacy, particularly in common cancers like breast cancer that show limited response to PD-L1/PD-1 targeted therapies [26, 27].

FDA approvals in 2019 and 2021 introduced PD-L1/PD-1 ICBs therapies for triple-negative breast cancer (TNBC) [28]. Despite TNBC’s favorable immunological profile [27, 29, 30], clinical responses remain modest. These therapies are now expanding to other breast cancer subtypes, including ER-positive and HER2-positive/negative tumors [26, 31]. Therefore, better understanding of PD-L1 biology, particularly its N-glycosylation, may improve patient selection and therapeutic outcomes for breast cancer immunotherapy.

To address these knowledge gaps, we investigated how each PD-L1 N-glycosylation site, individually and collectively, affects PD-L1 binding to PD-1 and the efficacy of clinical PD-L1/PD-1-targeting ICBs. By studying these interactions in both human TNBC and ER+ breast cancer models, we provide comprehensive insights into how PD-L1 N-glycosylation impacts two distinct breast cancer subtypes.

Here, we use PD-L1 glycosylation variants that we have recently developed [32]. These variants include single mutated PD-L1 at each of its four N-glycosylation sites, and a fully non-glycosylated PD-L1; the WT and mutated PD-L1 variants were overexpressed in two cellular systems (human TNBC MDA-MB231 cells and human MCF7 luminal-A ER+ breast tumor cells), and were co-cultured with PD-1 biosensor cells - designated Immune Checkpoint Artificial Reporter overexpressing PD-1 (IcAR-PD-1) [33, 34] - to assess the functional bioavailability of these PD-L1 glycosylation mutants. This method allowed us to examine in detail the way modifications at specific N-glycosylation sites influence the interactions of PD-L1 with PD-1, and to determine the roles of PD-L1 glycosylation in regulating the blocking efficacy of anti-PD-L1/anti-PD-1 ICBs. To further validate our findings in a physiological context, we conducted CD8+ T-cell activation and cytotoxicity assays using primary T-cells, providing insights into the impact of PD-L1 glycosylation states on a biologically relevant system.

Overall, this work is the first to demonstrate how N-glycosylation of PD-L1 influences its bioavailability and function. Furthermore, our work characterizes in detail the complex relationships between the glycosylation status of PD-L1 and the efficacy of PD-L1/PD-1-targeting ICBs.

## Methods

### Tissue culture

IcAR-PD-1 (Mouse BW5147 thymoma, ATCC TIB-47) cells were maintained in RPMI 1640 medium supplemented with 10% (v/v) fetal bovine serum (FBS), penicillin, streptomycin, glutamine, and sodium pyruvate (1 mM each). Human breast cancer cell lines MDA-MB231(ATCC HTB-26) and MCF7 (ATCC HTC-22), human melanoma cell line A375 (ATCC CRL-1619) and human non-small cell lung cancer cell line A549 (NSCLC; ATCC CCL-185) were transduced to express WT PD-L1 and all PD-L1 mutant variants as well as pQCXIP alone, as a control; the mutants were described in our previous publication ([32]; The current study demonstrates new flow cytometry validations). Of note, MDA-MB231 cells express PD-L1 endogenously, therefore the expression of endogenous PD-L1 was inhibited in the cells by CRISPR-Cas9 prior to infection with WT PD-L1, its N-glycosylation mutants or vector control. Cancer cell lines were cultured in Dulbecco’s Modified Eagle’s Medium (DMEM) containing 10% (v/v) FBS, along with penicillin, streptomycin, glutamine, and sodium pyruvate (1 mM each). MDA-MB231 and MCF7 cells were maintained with the addition of 1 µg/mL of puromycin (selection for PD-L1/sham-expressing vectors) and 200 µg/mL of G418 (selection for mCherry-expressing vector) (both from InvivoGen, CA, USA). All media and supplements were purchased from Sartorius (Göttingen, Germany). Cells were incubated at 37°C in a humidified incubator with 5% CO_2_.

### PD-L1 flow cytometry analyses

Cells were prepared for flow cytometry by washing them twice with a solution composed of phosphate-buffered saline (PBS), 0.05% sodium azide, and 1% fetal bovine serum (PAF). Cells were then counted and plated at 10^5^ cells/well in a 96-well plate. When required, adherent cells were detached using Versene Solution (catalog no. 15040066, Gibco), washed twice with PAF, and resuspended in PAF containing specific monoclonal antibodies. Commercial antibodies to PD-L1 included mouse anti-human CD274 (B7-H1, PD-L1) antibody (catalog no. 329702, Clone 29E.2A3, BioLegend, CA, USA, RRID: AB_940372); mouse IgG2b antibodies were used as isotype control (catalog no. 402202, BioLegend, RRID: AB_2936439). All clinical antibodies were acquired from Soroka University Medical Center (SUMC). Clinical anti-PD-L1 antibodies were Durvalumab (lot: PS15802), Avelumab (lot: AU36684), and Atezolizumab (lot: A2004016), with human IgG1 isotype antibody (catalog no. BE0297, Bio X Cell, NH, USA, RRID: AB_2687817) serving as control. PD-1 expression on IcAR cells was evaluated using clinical anti-PD-1 antibodies: Pembrolizumab (lot: U019110), Nivolumab (lot: AAV1063), and Cemiplimab (lot: 1F012F). Antibodies were added at a final concentration of 2 µg/mL, and samples were incubated for 45 min on ice. After washing, secondary antibodies were applied: Alexa Fluor 488-conjugated goat anti-mouse IgG (H + L) (catalog no. 115-545-062, Jackson ImmunoResearch Laboratories, PA, USA, RRID: AB_2338845) for commercial antibodies, and Alexa Fluor 488-conjugated goat anti-human IgG (H + L) (catalog no. 109-545-088, Jackson ImmunoResearch Laboratories, RRID: AB_2337838) for clinical antibodies. Samples were incubated for additional 45 min on ice, washed, and resuspended in PAF containing 4′,6-diamidino-2-phenylindole (1 µg/mL) as a viability marker. Samples were analyzed using a Beckman CytoFLEX flow cytometer. Isotype-matched controls were used to set appropriate gates, and samples were analyzed in biological triplicate for each marker. Cells expressing specific markers were reported as a percentage of total gated events.

### Detection of total-cellular PD-L1 expression in fixed cells

MDA-MB231 and MCF7 cells were fixed for 10 min at room temperature in 10 mL of 4% formaldehyde solution (catalog no. 6450323F1, Bio-Lab, Israel), followed by two washes with PBS. Fixed cells were then stained using two methods: surface staining without permeabilization and total-intracellular staining with permeabilization. For surface staining, fixed cells were incubated with the commercial anti-PD-L1 antibody (catalog no. 329702, BioLegend) at 2 µg/mL in PAF for 45 min at room temperature. After washing, cells were incubated with Alexa Fluor 488-conjugated goat anti-mouse IgG (H + L) secondary antibody (catalog no. 115-545-062, Jackson ImmunoResearch Laboratories, RRID: AB_2338845) for 45 min at room temperature. For intracellular staining, the True-Nuclear™ Transcription Factor Buffer Set (catalog no. 424401, BioLegend) was used according to the manufacturer’s instructions. Fixed cells were permeabilized for 45 min at room temperature using True-Nuclear™ 1X Perm Buffer (catalog no. 424401, BioLegend, CA, USA). The commercial anti-PD-L1 antibody was then added at 2 µg/mL in True-Nuclear™ 1X Perm Buffer and incubated for 45 min at room temperature. After washing, cells were incubated with the Alexa Fluor 488-conjugated goat anti-mouse IgG (H + L) secondary antibody in True-Nuclear™ 1X Perm Buffer for 45 min at room temperature. Cells were washed twice with True-Nuclear™ 1X Perm Buffer before resuspension in flow cytometry staining buffer for analysis.

### IcAR-PD-1 functional bioavailability assay

MDA-MB231 and MCF7 cells were seeded in flat 96-well plates at a density of 2.5 × 10^4^ cells/well and cultured for 24 h. For functional bioavailability assays, 1 × 10^5^ IcAR-PD-1 cells (at a density of 1 × 10^6^ cells/mL) were then added to each well. In titration experiments, cancer cells were co-cultured with IcAR-PD-1 cells at different concentrations (please see relevant figures). Co-cultures were incubated for 24 h at 37°C in a humidified incubator (5% CO_2_). Then, supernatants were collected and analyzed for mIL-2 production using a commercial sandwich ELISA kit according to the manufacturer’s instructions (please see more details below). This assay serves as reporter for PD-1 activation following PD-L1 binding, by measuring IL-2 production; such PD-1 activation, happening in the context of immune functions, leads eventually to T-cell suppression.

Experiments detecting the ability of anti-PD-L1 or anti-PD-1 ICBs to block PD-L1/PD-1 interactions were performed [1] using clinical anti-PD-L1 antibodies - Atezolizumab, Durvalumab and Avelumab - at concentrations ranging between 1.25 µg/mL to 20 µg/ml; [2] using clinical anti-PD-1 antibodies - Pembrolizumab, Nivolumab and Cemiplimab - at concentrations ranging between 1.25 µg/mL and 40 µg/mL. In blocking experiments with fixed cancer cells, a concentration of 10 µg/mL was used for all antibodies. Positive control wells were coated with 50 µL of Pembrolizumab (10 µg/mL) (data not shown), while negative control wells contained only media. The antibodies were added to cancer-IcAR-PD-1 co-cultures together with the IcAR-PD-1 cells for 24 h. At the end of this incubation period, cell supernatants were collected, and mIL-2 levels were determined by ELISA (as described below). When the IcAR-PD-1 system was used in the presence of anti-PD-L1 or anti-PD-1 ICBs, higher levels of IL-2 production indicate effective blocking of the PD-L1/PD-1 interaction, while lower levels suggest functional binding of PD-L1 to PD-1, resulting in T-cell inhibition.

### Supernatant collection for ELISA assays

Supernatants were collected from IcAR-PD-1 culture plates after 24 h, or from cancer cells after 48 h, for murine IL-2 detection. Supernatants were collected after 24–72 h from cancer cells, for PD-L1 detection. Cellular debris was removed by centrifugation at 1,000 g for 10 min. The clarified supernatants were concentrated using 30 kDa Amicon Ultra-15 centrifugal filter units (catalog no. UFC903096, Millipore, MA, USA) at 3,000 g for 15 min, resulting in a 1:15 concentration ratio.

### Murine IL-2 ELISA

IcAR-PD-1 activation was assessed by measuring mIL-2 production using a sandwich ELISA. Supernatants from co-cultures of IcAR-PD-1 and cancer cells were collected at times indicated above. The 96-well ELISA plates were coated overnight at 4°C with purified anti-mouse IL-2 antibody (Catalog no. 503702, BioLegend, RRID: AB_315292) diluted in coating buffer (0.1 M Na_2_HPO_4_, pH 9.0). After washing, the plates were blocked with 10% FBS in PBS containing 0.05% Tween 20 for 1 h at room temperature. Following another wash, the collected supernatants and mIL-2 standards were added to the wells and incubated for 2 h at room temperature. After washing again, biotinylated anti-mouse IL-2 detection antibody (Catalog no. 503804, BioLegend, RRID: AB_315298) was added and incubated for 1 h at room temperature. Subsequently, streptavidin-horseradish peroxidase conjugate (catalog no. 016-030-084, Jackson ImmunoResearch, PA, USA, RRID: AB_2337238) was added and incubated for 30 min. After a final wash, TMB substrate (catalog no. TMBW-0100-01, Surmodics, MN, USA) was added to each well. Absorbance was measured at 650 nm using a microplate reader.

### PD-L1 ELISA

High-binding 96-well plates were coated with Durvalumab (1 µg/mL, 70 µL/well) overnight at 4°C. After washing, plates were blocked with 250 µL of 2% BSA for 1.5 h at 37°C. Samples or standard (recombinant human PD-L1 starting at 500 ng/mL with 1:1 serial dilutions in PBS 1X) were added (50 µL/well) and incubated. Murine anti-human PD-L1 detection antibody (1 µg/mL, catalog no. 329702, BioLegend, RRID: AB_940372) was applied for 1 h at 37°C, followed by peroxidase-conjugated goat anti-mouse IgG (1 µg/mL, catalog no. 115-035-146, Jackson ImmunoResearch, RRID: AB_2307392). After a final wash, TMB substrate (as above) was added to each well. Absorbance was measured at 650 nm using a microplate reader.

### EV extraction

Supernatants were collected from MDA-MB231 and MCF7 cells cultured for 72 h and centrifuged at 400 g for 3 min to pellet cells. The supernatants were then centrifuged at 15,000 g for 30 min (Hanil high-speed centrifuge, Supra R22) to remove cell debris, followed by filtration through a 0.22 μm filter. For EV isolation, the filtered supernatants were ultracentrifuged at 100,000 g for 90 min using a Type 70 Ti rotor (Optima XL-80 K, Beckman, CA, USA). The resulting EV pellets were pooled and concentrated by a second round of ultracentrifugation at 100,000 g for 90 min. All supernatants discarded during EV isolation were combined and concentrated to obtain the sPD-L1 fraction.

### Cell lysate preparation

Adherent cancer cells were washed with cold PBS and harvested using versene. Cell pellets were resuspended in 350 µL of ice-cold RIPA buffer (without SDS) supplemented with protease inhibitors (1:100 dilution, Catalog no. P8340, Sigma-Aldrich, MO, USA). After 30 min incubation at 4°C, lysates were centrifuged at 14,000 g for 15 min at 4°C to pellet cell debris. The supernatants were collected for analysis.

### Peripheral mononuclear blood cell (PBMC) isolation

Primary T-cells were isolated from healthy consenting donors. To this end, blood (7 mL) was collected using a DG-veinset (VSET21) into K3EDTA tubes (Catalog no. 455036, Greiner Bio-One, Kremsmunster, Austria). The blood was diluted 1:1 with PBS containing 2% FBS and layered onto Ficoll-Paque (Catalog no. Cytiva 17144002, Thermo-Fisher Scientific, MS, USA) for density gradient centrifugation at 400 g for 30 min at room temperature. The mononuclear cell layer was carefully collected from the interphase and subjected to a second round of Ficoll-Plaque separation to enhance purity. After the second centrifugation, mononuclear cells were collected again from the interphase and washed twice with PBS containing 2% FBS. Cells were then suspended in RPMI to a final concentration of 2 × 10^6^ cells/mL with 10% human serum (Sigma-Aldrich) and 40 IU/mL recombinant huma IL-2 (rhIL-2, catalog no. 200-02, PeproTech, NJ, USA).

### Determining the activation potential of CD8+ T-cells

Tumor cells expressing WT PD-L1 or PD-L1 variants were seeded in a 96-well flat-bottom plate at a density of 2.5 × 10^4^ cells/well in 100 µL of DMEM, and incubated for 24 h at 37°C in a humidified CO_2_ incubator (5% CO_2_). After 24 h, peripheral blood mononuclear cells (PBMCs) were added to each well at a volume of 100 µL, containing 1.5 × 10^5^ cells, to achieve an effector-to-target (E: T) ratio of 3:1. The assay was performed in the presence of 1 µg/mL purified anti-human CD3 (hCD3) antibody (OKT3, catalog no. 300437, BioLegend, RRID: AB_11147760) and rhIL-2 (as above), to induce T-cell activation; FITC-conjugated anti-human CD107a antibody (catalog no. 328606, BioLegend, RRID: AB_1186036) were added as markers of T-cell activation/degranulation. The cell co-culture was incubated for 4 h at 37°C. For ICB function experiments, ICBs were added together with PBMCs, for a total incubation time of 4 h. Following incubation, cells were washed twice with PAF 1X and stained with antibodies to cell-surface markers (all from Biolegend, unless otherwise indicated): CD3 (APC, catalog no. 300439, RRID: AB_2562045), CD8 (APC/Fire™ 750, catalog no. 344746, RRID: AB_2572095), CCR7 (PE, catalog no. 353204, RRID: AB_10913813), CD45 (Krome Orange, catalog no. A96416, Beckman Coulter, CA, USA, RRID: AB_2888654), and PD-1 (PE-Cy7, catalog no. 621616, RRID: AB_2832836), to identify specific CD8+ T-cell subsets. Samples were incubated for 45 min on ice, washed, and resuspended in PAF 1X containing DAPI (1 µg/mL) as a viability marker. For the killing assay, target cancer cells were detached using Versene solution, washed twice with PAF 1X, and resuspended in PAF 1X containing DAPI (1 µg/mL) as a viability marker. For each donor, a positive control (PC) was included by adding OKT3 without target cells to assess maximal activation potential, while a negative control (NC) consisted of media only, without OKT3, to account for background activity. Samples were analyzed using a Beckman CytoFLEX flow cytometer. Each condition was tested in triplicate biological repeats to ensure reproducibility. Cells expressing specific markers were reported as a percentage of total gated events to minimize false positives.

### Statistical analyses

Statistical analyses were conducted using GraphPad Prism v10.3. Data are presented as mean ± standard deviation (SD) or median with interquartile range (IQR), depending on the dataset characteristics. For comparisons between multiple groups, one-way ANOVA was used, followed by Bonferroni or Dunnett’s post-hoc tests to identify specific group differences. Welch’s ANOVA was applied for datasets with unequal variances, as determined by Levene’s Test (*p* < 0.05), which assesses the equality of variances across groups. Two-way ANOVA was employed for experiments involving multiple factors to evaluate main effects and interactions. Multiple paired t-tests were used to compare paired data points, with corrections for multiple comparisons applied as needed. Statistical significance was indicated as follows: *p < 0.05, **p < 0.01, ***p < 0.001, ****p < 0.0001. All experiments were performed in triplicate biological repeats or with multiple donors to ensure reproducibility and reliability of the results.

## Results

### The ability of PD-L1 to induce PD-1 functions is independent of any single N-glycosylation site of PD-L1

**Fig. 1:**
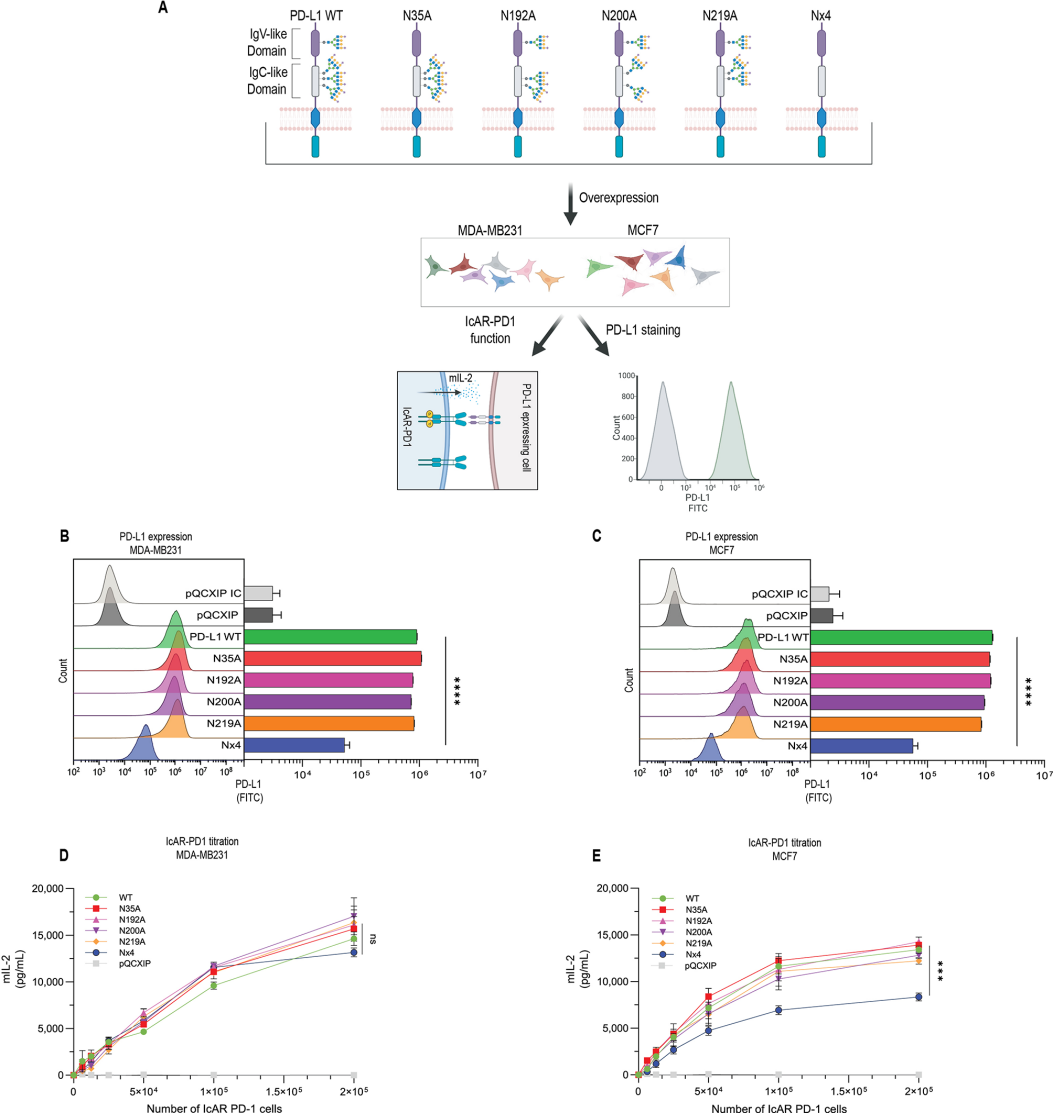
PD-L1 ability to interact with PD-1 is independent of any single N-glycosylation site of PD-L1. **(A)** Schematic representation of the structure of PD-L1 and its glycosylation mutants. The scheme highlights the sites of mutation/s in different glycosylation PD-L1 variants, as well as the IcAR-PD-1 functional assay and cell staining procedures. **(B)** PD-L1 expression in MDA-MB231 cells. Left: A representative histogram of PD-L1 expression using a commercial anti-PD-L1 antibody. Isotype control (IC) is demonstrated for pQCXIP-expressing cells (vector control); IC was negative also for all other cell types (data not shown). Right: Bar chart quantifying expression levels, where data represent mean ± SEM from three independent experiments. Welch’s ANOVA was then performed to compare group means, followed by Games-Howell post-hoc test for pairwise comparisons. Significant differences from WT PD-L1 are indicated. **(C)** PD-L1 expression in MCF7 cells. Left: Histogram of PD-L1 staining. Right: Bar chart of expression levels. Data analysis and statistics as in panel B. **(D)** Functional assays with IcAR-PD-1 cells on MDA-MB231 cells expressing WT PD-L1 and PD-L1 glycosylation mutants. Titration of IcAR-PD-1 cells against a constant number of MDA-MB231 cells expressing WT PD-L1 or different PD-L1 mutated variants. Data points represent mean ± SEM from triplicate biological experiments. One ANOVA with Bonferroni correction was used to assess statistical significance between PD-L1_Nx4_ and PD-L1_WT_. No statistical differences were noted between cells expressing the different PD-L1 variants and WT PD-L1-expressing cells. **(E)** Functional assays with IcAR-PD-1 cells on MCF7 mutants. Titration assay as in panel D, performed on MCF7 cells with PD-L1_WT_ and PD-L1 mutations. The difference between PD-L1_WT_ PD-L1_Nx4_-expressing cells was significant. ***p < 0.001, ****p < 0.0001, ns = not significant

The first aim of this study was to determine whether post-translational N-glycosylation of PD-L1 influences its interactions with PD-1 and activates PD-1-mediated functions. To this end, we used a PD-1 biosensor cellular system, designated Immune Checkpoint Artificial Reporter overexpressing PD-1 (IcAR-PD-1). In this model, production of mIL-2 serves as a readout for the functional interactions between PD-1 and its ligands - mimicking the ability of PD-1 to induce immune suppression in T-cells (Fig. 1A).

Using the IcAR-PD-1 system, we took advantage of the MDA-MB231 and MCF7 breast cell lines carrying mutations in PD-L1 N-glycosylation sites, as presented in our previous study [32]. Specifically, each of the two cell lines was transduced to overexpress WT PD-L1 or a single glycosylation site mutation: N35A, N192A, N200A, and N219A. In addition, a PD-L1 variant aberrant in all N-glycosylation sites - named Nx4 (all four N-glycosylation sites were replaced by alanine) - was expressed in both cell types (Fig. 1A).

Prior to determining the functionality of each of the PD-L1 variants, we assessed their surface expression levels. Both cell lines were stained using a commercial anti-PD-L1 antibody, and PD-L1 expression was analyzed by flow cytometry (Fig. 1B and C). No detectable PD-L1 expression was observed in both cell lines transduced with the expression vector alone (pQCXIP). In contrast, high and similar expression levels of PD-L1 were observed in the WT and each of the single mutants of PD-L1 (N35A, N192A, N200A, N219A). Notably, the expression of the Nx4 variant was significantly lower than that of the WT protein (Welch’s ANOVA, *p* < 0.0001 for both cell lines; the expression levels demonstrated herein agree with our previous report [32]). Specifically, the Nx4 PD-L1 variant exhibited a 17-fold decrease in MDA-MB231 cells and a 22-fold decrease in MCF7 cells compared to their respective WT protein. Similar results are shown in staining tumor cells with clinically approved anti-PD-L1 antibodies: Durvalumab, Avelumab, and Atezolizumab (Supplementary Fig. 1A1-6 for MDA-MB231 cells and B1-6 for MCF7 cells). Additionally, PD-L2 staining showed no expression in MDA-MB231 cells and low levels in MCF7 cells (Supplementary Fig. 1C1 and C2, respectively).

Next, we explored the functional bioavailability of WT PD-L1 and of each PD-L1 mutant, namely their ability to induce signaling through PD-1, resulting in the production of mIL-2 in the IcAR-PD-1 system. To this end, we co-cultured tumor cells expressing PD-L1_WT_ or different PD-L1 variants with increasing amounts of IcAR-PD-1 cells. After 24 h of co-culture, we measured mIL-2 as a readout for PD-L1 functionality (Fig. 1D and E). In both MDA-MB231 and MCF7 cells, cells transfected with the control vector alone (pQCXIP) elicited no response, as expected; cells overexpressing PD-L1_WT_ and single-mutated PD-L1 showed a strong ability to induce mIL-2 production through interaction with PD-1, at similar levels.

However, when the PD-L1_Nx4_ mutant lacking all N-glycosylation sites was explored, we found that it had a lower ability to induce mIL-2 production in MCF7 cells (Fig. 1E); these findings are in line with the reduced membrane expression of this mutant (One-way ANOVA, *p* < 0.001) (Fig. 1C). In contrast, in MDA-MB231 cells the PD-L1_Nx4_ mutant manifested similar phenotype to PD-L1_WT_ and the other mutants in its ability to interact with PD-1 (Fig. 1D), despite its lower expression levels at the cell surface of the cells (Fig. 1B). The observations made in MDA-MB231 cells suggest that the functional interaction between PD-L1 mutants and IcAR-PD-1 cells may not directly correlate with surface expression levels observed by flow cytometry.

### PD-L1 N35 is required for optimal efficacy of anti-PD-L1 in blocking the interactions between PD-L1 and PD-1, whereas PD-L1 N-glycosylation at all four sites interferes with this blocking activity

**Fig. 2:**
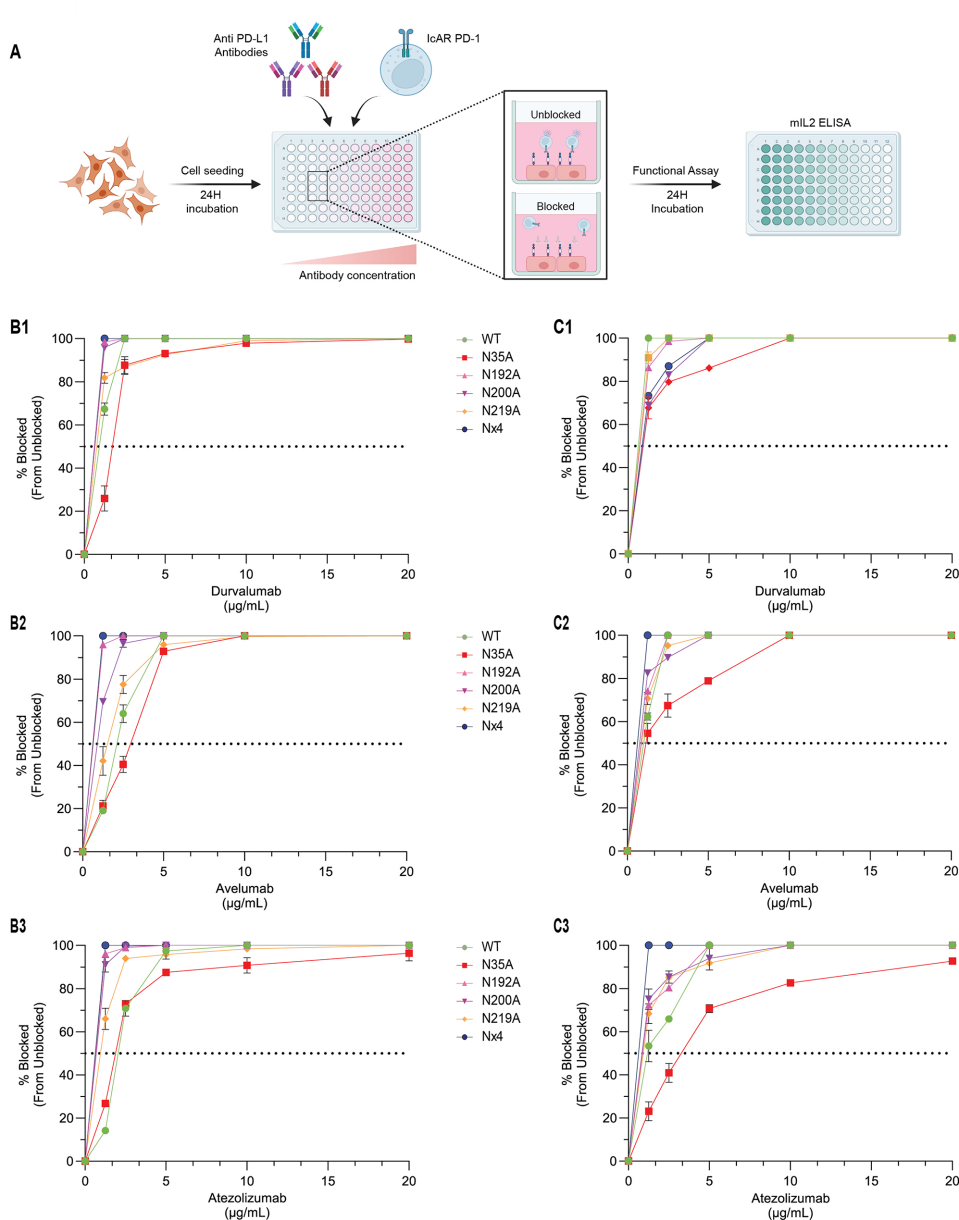
The PD-L1 N35A variant perturbs the ability of anti-PD-L1 ICBs to block PD-L1/PD-1 interactions, whereas the lack of glycosylation at all four PD-L1 glycosylation sites enhances blocking. **A.** Schematic representation of the experimental procedure determining the efficacy of anti-PD-L1 ICBs in blocking the interactions between PD-L1_WT_/different PD-L1 glycosylation variants and PD-1. Here, PD-L1-expressing cells were incubated with increasing concentrations of anti-PD-L1 antibodies (Atezolizumab, Avelumab or Durvalumab). Then, IcAR-PD-1 cells were added to assess PD-L1/PD-1 binding inhibition, measured by the levels of mIL-2 (determined by ELISA). **B**. The capacity of anti-PD-L1 antibodies in blocking the interactions of PD-L1 (WT and glycosylation variants) expressed by MDA-MB231 cells with PD-1. Graphs demonstrate the normalized blocking capacity of Atezolizumab (B1), Avelumab (B2), and Durvalumab (B3) on each cell type. Antibody concentrations ranged from 1.5 µg/mL to 20 µg/mL. Data points represent mean ± SEM from triplicate biological repeats. **C.** The capacity of anti-PD-L1 antibodies in blocking the interactions of PD-L1 (WT and glycosylation variants) expressed by MCF7 cells with PD-1. As in panel B, the graphs display the normalized blocking capacity of Atezolizumab (C1), Avelumab (C2), and Durvalumab (C3). The same antibody concentration range was used

Next, we assessed the effect of PD-L1 N-glycosylation on the blocking capacity of anti-PD-1 treatments. We used a co-culture system with the different types of tumor cells and IcAR-PD-1 cells, in the presence or absence of antibodies to block PD-L1. We tested three FDA-approved PD-L1 blockers - Durvalumab [35], Avelumab [36], and Atezolizumab [37] - at concentrations ranging from 1.5 µg/mL to 20 µg/mL. Then, we measured the extent of PD-L1/PD-1 interaction by quantifying mIL-2 levels using ELISA (Fig. 2A).

In both MDA-MB231 and MCF7 cell lines, the anti-PD-L1 antibodies effectively blocked the interactions between PD-L1 and PD-1, whether PD-L1 was in its WT form or with N192A, N200A, or N219A mutations. Most PD-L1 variants exhibited similarly high blocking patterns with all antibodies tested; however, the PD-L1_N35A_ mutant stood out as an exception (Fig. 2). In contrast to the other variants, when PD-L1 was mutated at N35, the antibodies generally demonstrated lower blocking efficacy. In MDA-MB231 cells (Fig. 2B1-B3 and Supplementary Fig. 2A1-A6), the PD-L1_N35A_ mutation moderately reduced the effectiveness of all anti-PD-L1 antibodies in blocking the PD-L1/PD-1 interaction. Higher antibody concentrations were required to achieve complete blocking compared to PD-L1_WT_. In MCF7 cells (Fig. 2B1-B3 and Supplementary Fig. 2B1-B6), the blocking efficacy of Avelumab and Atezolizumab was substantially reduced in PD-L1_N35A_. These findings indicate that the integrity of the N35 glycosylation site is crucial for optimal inhibitory potency of antibodies targeting PD-L1.

A particularly intriguing finding was that when all N-glycosylation sites of PD-L1 were impaired in the PD-L1_Nx4_ mutant, the inhibitory capacity of anti-PD-L1 antibodies was enhanced compared to cells expressing WT PD-L1. Notably, this increased activity of the PD-L1_Nx4_ mutant was observed despite its lower surface expression in both MDA-MB231 and MCF7 cells. Area under the curve (AUC) analysis further accentuated the differences between PD-L1_WT_, the PD-L1_N35A_ mutant, and PD-L1_Nx4_ mutant (Supplementary Fig. 2C and 2D). These findings demonstrate that N-glycosylation of PD-L1 at all four sites disrupts the ability of anti-PD-L1 ICBs to interfere with PD-L1 binding to PD-1.

### PD-L1 N-glycosylation sites regulate the efficacy of anti-PD-1 functions, with N35 being required for optimal inhibitory potency and full N-glycosylation inhibiting it

**Fig. 3:**
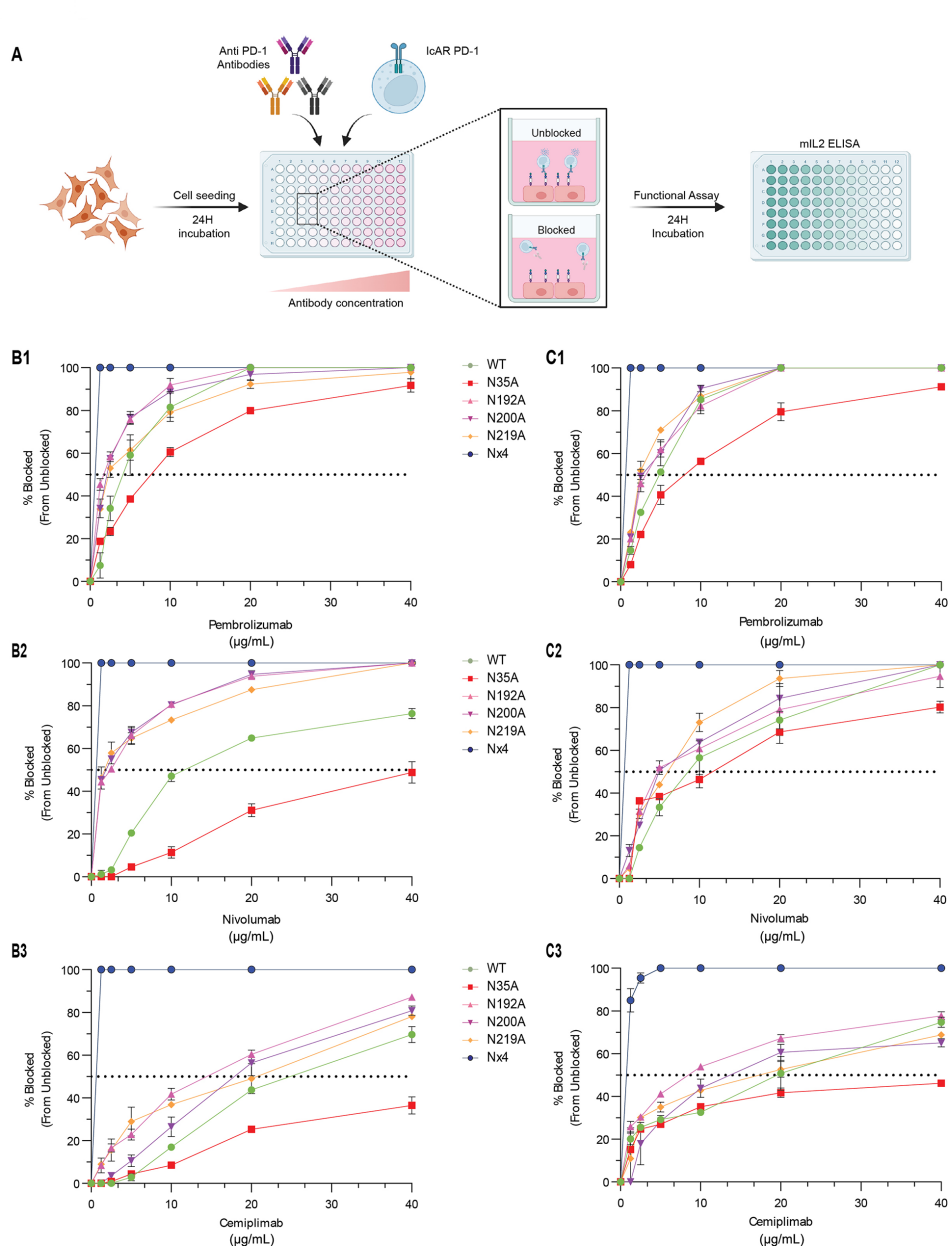
The PD-L1 N35A variant perturbs the ability of anti-PD-1 ICBs to block PD-L1/PD-1 interactions, whereas the lack of glycosylation at all four PD-L1 glycosylation sites enhances blocking. **A.** Schematic representation of the experimental procedure determining the efficacy of anti-PD-1 ICBs in blocking the interactions between WT PD-L1/different PD-L1 glycosylation variants and PD-1. Here, PD-L1-expressing cells were incubated with increasing concentrations of anti-PD-1 antibodies (Pembrolizumab, Nivolumab, Cemiplimab). Then, IcAR-PD-1 cells were added to assess PD-L1/PD-1 binding inhibition, measured by the levels of mIL-2 (determined by ELISA). **B**. The capacity of anti-PD-1 antibodies in blocking the interactions of PD-L1 (WT and glycosylation variants) expressed by MDA-MB231 cells with PD-1. Graphs demonstrate the normalized blocking capacity of Pembrolizumab (B1), Nivolumab (B2), and Cemiplimab (B3) on each cell type. Antibody concentrations ranged from 1.5 µg/mL to 40 µg/mL. Data points represent mean ± SEM from triplicate biological repeats. **C.** The capacity of anti-PD-1 antibodies in blocking the interactions of PD-L1 (WT and glycosylation variants) expressed by MCF7 cells with PD-1. As in panel B, the graphs display the normalized blocking capacity of Pembrolizumab (C1), Nivolumab (C2), and Cemiplimab (C3). The same antibody concentration range was used

We then investigated the effect of mutation of PD-L1 glycosylation sites on the blocking capacity of clinical anti-PD-1 antibodies by measuring the inhibition of mIL-2 readout signals generated when PD-L1 interacts with PD-1. To achieve this, we co-cultured tumor cells expressing the PD-L1_WT_ or different PD-L1 variants with IcAR-PD-1 cells in the presence and absence of increasing concentrations (1.5 µg/mL to 40 µg/mL) of FDA-approved anti-PD-1 antibodies: Pembrolizumab [38], Nivolumab [39], and Cemiplimab [39]; these antibodies bind well to PD-1 on IcAR-PD-1 cells (Supplementary Fig. 3A). We assessed the ability of these antibodies to block PD-L1/PD-1 interactions across our panel of PD-L1 glycosylation mutants, as indicated by mIL-2 levels (Fig. 3A).

Titration of clinical anti-PD-1 antibodies against MDA-MB231 cells and MCF7 cells expressing PD-L1 mutants revealed distinct patterns across cell lines and antibodies (Fig. 3B1-B3 for MDA-MB231 cells and 3C1-C3 for MCF7 cells; Supplementary Fig. 3B and 3C). In cells expressing PD-L1_WT_, Pembrolizumab exhibited the highest blocking capability compared to Nivolumab and Cemiplimab in both the MDA-MB231 and MCF7 cell lines. Notably, in both cell lines, pembrolizumab completely blocked the PD-L1/PD-1 functional interaction induced by WT PD-L1 at a concentration of 20 µg/mL (Fig. 2C1 and 3C1).

Generally, the PD-1-blocking capacities of all three anti-PD-1 antibodies were enhanced when the tumor cells expressed the N192A, N200A, or N219A PD-L1 glycosylation mutants. This indicates that when PD-L1 is glycosylated at these sites, anti-PD-1 antibodies have a reduced ability to block PD-L1/PD-1 interactions. Moreover, when the cancer cells expressed PD-L1_Nx4_, all three antibodies demonstrated approximately 100% blocking capacity across all concentrations, starting at concentration as low as 1 µg/mL. AUC analyses confirmed this trend (Supplementary Fig. 3D and 3E), with PD-L1_Nx4_ showing significantly higher AUC values compared to PD-L1_WT_ across all antibody concentrations in both cell lines (one-way ANOVA, *p* < 0.0001 for all tested anti-PD-1 antibodies). These findings indicate that N-glycosylation of PD-L1 at all four sites interferes with the ability of anti-PD-1 antibodies to inhibit the interactions between PD-L1 and PD-1.

In contrast to PD-L1_Nx4_ mutant and the single mutants at the N192, N200, and N219 glycosylation sites, the anti-PD-1 antibodies consistently exhibited inferior blocking capacity when tested against cells expressing PD-L1_N35A_ mutant, compared to those expressing PD-L1_WT_. This observation was consistent across both cell lines and all clinical antibodies tested. Specifically, in MDA-MB231 cells, PD-L1_N35A_ mutant reached only 50% blocking of PD-1 activities at the highest concentration of Nivolumab (Fig. 3B2) and less than 50% with the highest Cemiplimab concentration (Fig. 3B3). In these cells, Pembrolizumab also demonstrated a lower ability to block PD-L1/PD-1 interactions, as evidenced by higher antibody concentrations required to reach 50% inhibition, compared to PD-L1_WT_. While PD-L1_N35A_ performed slightly better in MCF7 cells (Fig. 3C1-C3), surpassing the 50% blocking mark with Nivolumab (Fig. 3C2), it reached this threshold with Cemiplimab only at the highest concentration (Fig. 3C3). These results indicate that impairment of N35 glycosylation significantly reduced the efficacy of clinical antibodies in blocking PD-L1/PD-1 interactions.

To validate our initial observations, we performed additional experiments using two clinically relevant human cell lines: A375 melanoma cells and A549 NSCLC cells, both of which are commonly treated with anti-PD-L1 and anti-PD-1 ICBs [40]. We transduced these cells with vectors encoding PD-L1_WT_, PD-L1_N35A_, or PD-L1_Nx4_ variants and repeated the experiments described above. PD-L1 cell surface expression levels and the ability to activate the IcAR cell system were consistent with those observed in breast cancer cells (Supplementary Figs. 4A and 4B, respectively). Moreover, assays using anti-PD-L1 and anti-PD-1 ICBs confirmed the findings from MDA-MB231 and MCF7 cells, showing lower blocking activity of the ICBs in the presence of the PD-L1_N35A_ mutant and higher blocking efficacy with the PD-L1_Nx4_ mutant, compared to cells expressing WT PD-L1. The results, shown in Supplementary Fig. 4, further validate the patterns observed in flow cytometry, IcAR responses, and the blocking capacity of both anti-PD-L1 and anti-PD-1 ICBs.

Thus, in line with the importance of the N35 site in regulating the inhibitory capacity of all clinical anti-PD-L1 antibodies, the integrity of the N35 glycosylation site was found to be required for all three clinical anti-PD-1 antibodies to reach maximal inhibitory potency in A375 and A549 cells (Supplementary Fig. 3D and E). Our findings further demonstrate that PD-L1 glycosylation at all four sites impairs not only the ability of anti-PD-L1 but also of anti-PD-1 ICBs to block PD-L1 interactions with PD-1.

Overall, our investigation demonstrates that PD-L1 glycosylation sites significantly influence the blocking capacity of clinical anti-PD-L1 and anti-PD-1 antibodies. These findings suggest an underlying mechanism by which glycosylation affects the interaction between PD-L1, PD-1, and therapeutic antibodies.

### The levels of soluble PD-L1 are elevated in the lack of glycosylation at the N35 site, whereas the ablation of glycosylation at all four sites reduces the levels of the soluble protein

**Fig. 4:**
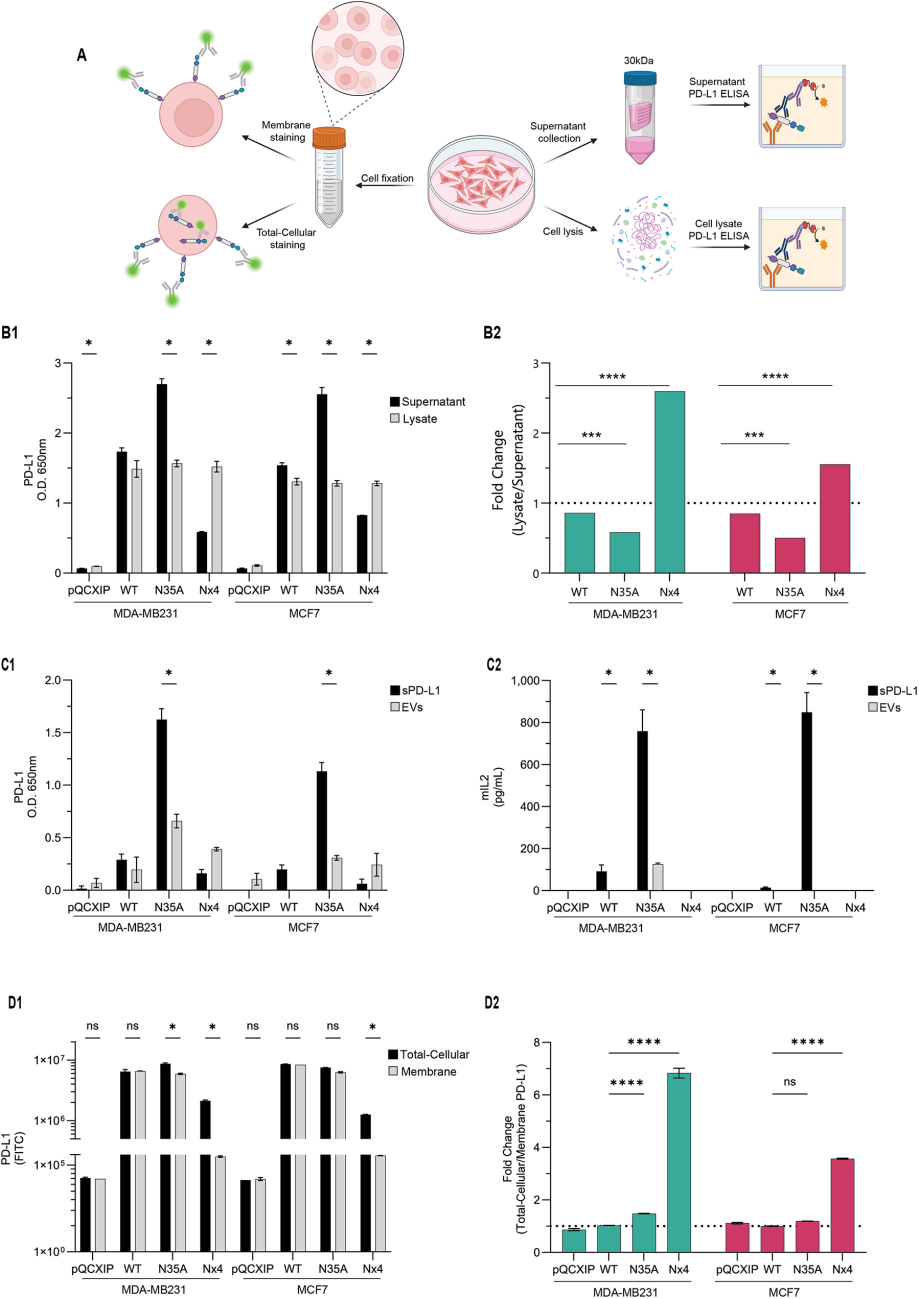
PD-L1_N35A_ is highly present in a soluble form in cell supernatants, whereas the fully non-glycosylated PD-L1 is present in only minimal levels in a soluble form. **A**. Schematic representation of the experimental procedure determining PD-L1 total-cellular levels in cell lysates and sPD-L1 levels in cell supernatants (by ELISA); in parallel, membrane and total-cellular PD-L1 levels were determined in paraformaldehyde-fixed cells. **(B)** PD-L1 levels in cell lysates and in 24-hour supernatants. (B1) PD-L1 levels were determined by ELISA assays, demonstrating PD-L1 levels in y-axis. Bars show mean values ± SEM from three independent experiments in MDA-MB231 and MCF7 cells. Multiple paired t-tests assessed differences between lysate and supernatant PD-L1 levels for each variant. (B2) Ratio of total-cellular to membrane PD-L1 levels. Bar graph showing the fold-change of total-cellular to membrane PD-L1 staining for PD-L1_WT_-expressing cells, and N35A and Nx4 PD-L1 mutants in MDA-MB231 and MCF7 cells. The y-axis represents the ratio of total-cellular to membrane PD-L1 staining intensity, based on the findings presented in panel B1. Bars show mean ± SEM from three independent experiments. Ordinary one-way ANOVA with Dunnett’s test compared PD-L1_WT_ to PD-L1 mutants N35A and Nx4. **(C)** PD-L1 levels in soluble fraction. (C1) PD-L1 levels of the EV-bound PD-L1 and sPD-L1 fractions of 72-hour supernatants were determined by ELISA assays, demonstrating PD-L1 concentrations in y-axis. Bars show mean values ± SEM from three independent experiments in MDA-MB231 and MCF7 cells. Multiple paired t-tests assessed differences between lysate and supernatant PD-L1 levels for each variant. (C2) Determining the ability of EV-bound PD-L1 and sPD-L1 to induce PD-1 functions, using the IcAR-PD-1 system. **(D)** Total-cellular vs. membrane PD-L1 levels. (D1) Total-cellular and membrane PD-L1 levels were determined for WT, N35A and Nx4 PD-L1-expressing MDA-MB231 and MCF7 cells by flow cytometry. Y-axis represents PD-L1 staining intensity. Bars show mean values ± SEM of triplicate biological repeats. Multiple paired t-tests assessed differences between total-cellular and membrane staining for each cell type. (D2) Bar graph illustrating the fold-change of total-cellular to membrane PD-L1 staining for each PD-L1 mutant. Y-axis shows the fold-change ratio. *p < 0.05, ***p < 0.001, ****p < 0.0001. NS = Not significant

Given that PD-L1_WT_ and PD-L1_N35A_ have similar surface PD-L1 levels (Fig. 1) and that PD-L1_N35A_ mutant impairs the blocking activities of anti-PD-L1 and anti-PD-1 (Figs. 2 and 3), we hypothesized that soluble PD-L1 (sPD-L1) might interfere with this interaction, reducing the effectiveness of ICBs by competing with their ability to bind to cell surface PD-L1 or to more strongly induce PD-1 functions. To investigate this hypothesis, we employed ELISA assays to detect sPD-L1 levels in cell supernatants and lysates, as well as flow cytometry to assess membrane-bound and total-cellular PD-L1 levels (Fig. 4A).

In both MDA-MB231 and MCF7 cells, we observed similar levels of PD-L1 in cell lysates of the different PD-L1 variants (Fig. 4B1). However, the levels of sPD-L1 in the supernatants were significantly higher for PD-L1_N35A_ cells compared to PD-L1_WT_ (multiple paired t-tests, *p* < 0.05). The ratio of total PD-L1 (in cell lysates) to sPD-L1 (in supernatants) decreased by approximately 35% in MDA-MB231 PD-L1_N35A_ cells and about 45% in MCF7 PD-L1_N35A_ cells compared to their PD-L1_WT_ counterparts. This reduction primarily resulted from increased sPD-L1 levels rather than changes in total-cellular PD-L1 levels (Fig. 4B2).

Moreover, we aimed to determine whether PD-L1 exists in extracellular vesicles (EVs) and to assess the relative content of PD-L1 in EVs compared to its soluble protein form (Fig. 4C1). To achieve this, we applied a series of centrifugation and ultracentrifugation protocols to separate the sPD-L1 fraction from the EV-bound PD-L1 fraction. The data in Fig. 4C1 demonstrate that PD-L1 is indeed present in EVs, but at lower levels than in its soluble form. Importantly, similar to sPD-L1, the levels of PD-L1_N35A_ in EVs were higher than those of PD-L1_WT_ (multiple paired t-tests, *p* < 0.05).

Further analyses demonstrate that the sPD-L1 form of PD-L1_N35A_ was much more effective than its EV form in activating PD-1, observed by mIL-2 levels produced by the IcAR-PD-1 cell system (Fig. 4C2). These findings suggest that the high soluble levels of PD-L1 produced by the PD-L1_N35A_ mutant might activate PD-1 and thus reduce the ability of antibodies to PD-1 to block the functions of PD-1 (as seen in Fig. 3). These findings explain well the reduced efficacy of anti-PD-1 antibodies in blocking PD-1 functions, as previously noted (Fig. 3).

PD-L1_Nx4_ mutant exhibited markedly reduced sPD-L1 levels, leading to an apparent increase in the lysate-to-supernatant ratio of 270% in MDA-MB231 cells and 150% in MCF7 cells. It is important to note that this increase in the ratio for PD-L1_Nx4_ mutants is due to decreased sPD-L1 levels rather than increased intracellular retention, as the total amount of PD-L1 in cell lysates remained similar to PD-L1_WT_ levels. These findings indicate that PD-L1_Nx4_ mutant enhances ICB-mediated blocking of PD-L1/PD-1 interactions by releasing lower levels of sPD-L1, reducing competition with ICBs and improving their efficacy (Figs. 2 and 3). However, contrary to this mechanism, sPD-L1 from PD-L1_Nx4_ cells did not induce lower mIL-2 production in IcAR-PD-1 reporter cells compared to sPD-L1 from PD-L1_WT_ cells (Fig. 4C2).

Next, we determined the total-cellular and membrane-bound levels of PD-L1_WT_, PD-L1_N35A_, and PD-L1_Nx4_ (Fig. 4D). In both MDA-MB231 and MCF7 cells, the levels of total-cellular and membrane-bound PD-L1 were generally comparable between PD-L1_WT_ and PD-L1_N35A_ cells (Fig. 4D1 and D2); a slight increase in total-cellular PD-L1 levels was observed in PD-L1_N35A_ mutant MDA-MB231 cells (Fig. 4D2; multiple paired t-tests, *p* < 0.05). The most significant change occurred in the PD-L1_Nx4_ mutant, where both MDA-MB231 and MCF7 cells displayed significantly elevated total-cellular compared to membrane-bound PD-L1 levels (multiple paired t-tests, *p* < 0.05). In MDA-MB231 cells, this resulted in a six-fold increase in the ratio of total-cellular to membrane-bound PD-L1 levels (Fig. 4D1). A similar trend was seen in MCF7 cells, where total-cellular PD-L1 levels were threefold higher than extracellular levels (Fig. 4D2). These results point to impaired trafficking or membrane localization in PD-L1_Nx4_ variant, potentially impacting its functional availability on the cell surface.

### PD-L1 N-glycosylation regulates ICB functions also when the protein is expressed by fixed cancer cells

**Fig. 5:**
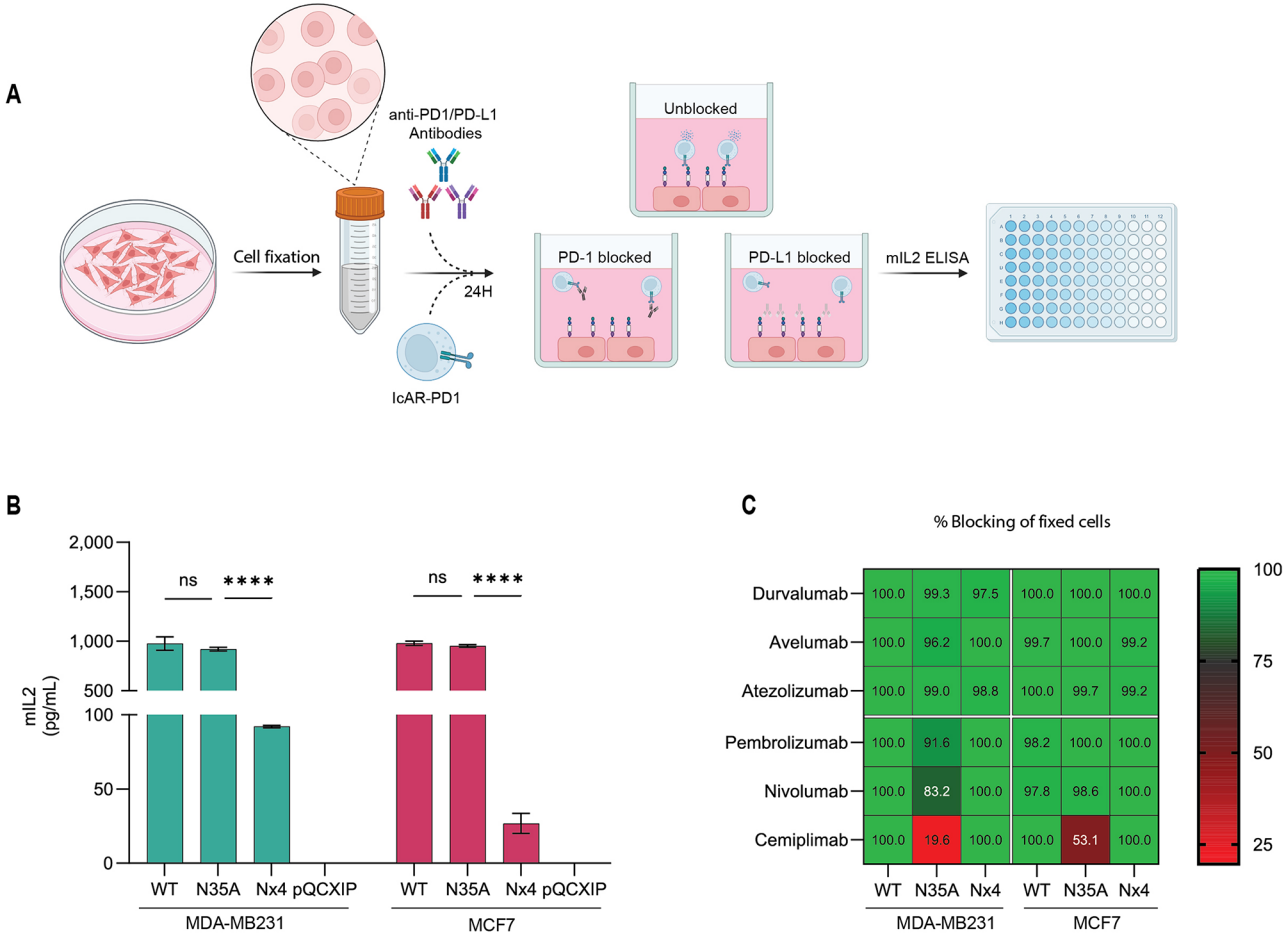
When Cemiplimab acts to inhibit the interactions of PD-L1 in fixed cells with PD-1, the N35A mutation limits Cemiplimab efficacy. **(A)** Schematic representation of the experimental procedure determining the ability of anti-PD-L1 and anti-PD-1 ICBs to block PD-L1/PD-1 interactions. When fixed MDA-MB231 and MCF7 cells - expressing WT PD-L1, N35A PD-L1 and Nx4 PD-L1 - were incubated with IcAR-PD-1 cells. The ICBs included anti-PD-L1 antibodies (Durvalumab, Avelumab, and Atezolizumab) and anti PD-1 antibodies (Pembrolizumab, Nivolumab and Cemiplimab). **(B)** IcAR-PD-1 response to fixed cells (Unblocked). Bar graph showing the baseline IcAR-PD-1 response (demonstrated by mIL-2 levels) to fixed cells expressing WT PD-L1 or PD-L1 glycosylation variants, without antibody blockade. pQXXIP represents target cells transduced with control backbone vector (negative control). Bars represent mean ± SEM of triplicate biological repeats. One-way ANOVA was performed to compare responses across mutations. **(C)** Antibody blocking efficiency on fixed cells. Normalized heat map representing the percentage of PD-L1/PD-1 interaction blocked by each antibody on fixed cells expressing PD-L1_WT_ or different PD-L1 variants. ****p < 0.0001. ns = Not significant

We set out to further explore the unique properties of the PD-L1 variants of interest (WT, N35A, and Nx4). To achieve this, we conducted an IcAR-PD-1 functional assay using formalin-fixed cancer cells as targets (Fig. 5A). Fixing the cells ‘froze’ their state at a specific moment, setting the number of PD-L1 molecules on the membrane and stopping any further intracellular trafficking. Fixing the cells also washed away soluble PD-L1 as a factor, allowing us to focus exclusively on interactions between membrane-bound PD-L1, PD-1, and blocking antibodies.

Thus, we incubated fixed cancer cells with IcAR-PD-1 cells for 24 h. The IcAR-PD-1 response to PD-L1_WT_ was high in both cell lines (Fig. 5B), indicating that PD-L1 expressed by fixed PD-L1_WT_ cells keeps its integrity and ability to activate PD-1. Fixed cancer cells expressing PD-L1_N35A_ had similar potency to cells expressing PD-L1_WT_ in inducing PD-1 functions, as was observed when cultured cell lines were used (Fig. 1). Fixed PD-L1_Nx4_ mutant cells did elicit some response as compared to control cancer cells transduced with the backbone vector. However, this was a significantly lower response compared to PD-L1_WT_ in both cell lines (one-way ANOVA, *p* < 0.0001). Specifically, the response was reduced by 10-fold in PD-L1_WT_ MDA-MB231 cells and by 22-fold compared to PD-L1_WT_ MCF7 cells (Fig. 5B).

We then evaluated the ability of clinical PD-L1/PD-1 ICBs (at a concentration of 10 µg/mL) to block PD-L1/PD-1 interactions when the cancer cells were fixed, therefore not giving rise to sPD-L1 presence in the cell media. We tested Durvalumab, Avelumab, and Atezolizumab against PD-L1, while Pembrolizumab, Nivolumab and Cemiplimab were tested against PD-1. The results were analyzed as a percentage of blocking, normalized vs. the values of unblocked controls, indicating the reduction in mIL-2 secretion in response to checkpoint blockade (Fig. 5C). Raw mIL-2 data are presented in Supplementary Fig. 5.

PD-L1_WT_ and PD-L1_Nx4_ variant were completely blocked by all antibodies at this concentration. In contrast, the anti-PD-1 blockade of the PD-L1_N35A_ mutant was incomplete in both cell lines. Cemiplimab failed to fully block the PD-L1/PD-1 interaction when N35 glycosylation was impaired in the PD-L1_N35A_ mutant (19.6% for MDA-MB231 and 53% blocking for MCF7). Additionally, discrepancies arose with Pembrolizumab and Nivolumab: MDA-MB231 PD-L1_N35A_ cells were not fully blocked compared to MCF7 PD-L1_N35A_ cells (91.6% vs. 100% with Pembrolizumab and 83.2% vs. 98.6% with Nivolumab).

Overall, according to findings with non-fixed cells (Figs. 2 and 3), the current analysis employing fixed cells revealed that the glycosylation site N35 accounted for most of the blocking abilities of the various anti-PD-1 antibodies. However, the PD-L1_N35A_ variation restricted the antibodies’ blocking functions more in non-fixed cells with high sPD-L1 levels than in fixed cells. Taken together, our data support the hypothesis that sPD-L1, which is highly expressed in non-fixed PD-L1_N35A_ cells, plays a role in reducing the blocking capacities of anti-PD-L1 and anti-PD-1 antibodies. Furthermore, Cemiplimab’s limited blocking despite the elimination of sPD-L1 suggests that intra-protein interaction may alter the protein’s properties and contribute to its resistance to ICBs.

### PD-L1 N-glycosylation, at all four sites, reduces the efficacy of ICBs in blocking PD-L1 interactions with PD-1 in anti-hCD3 activated T-cells

**Fig. 6:**
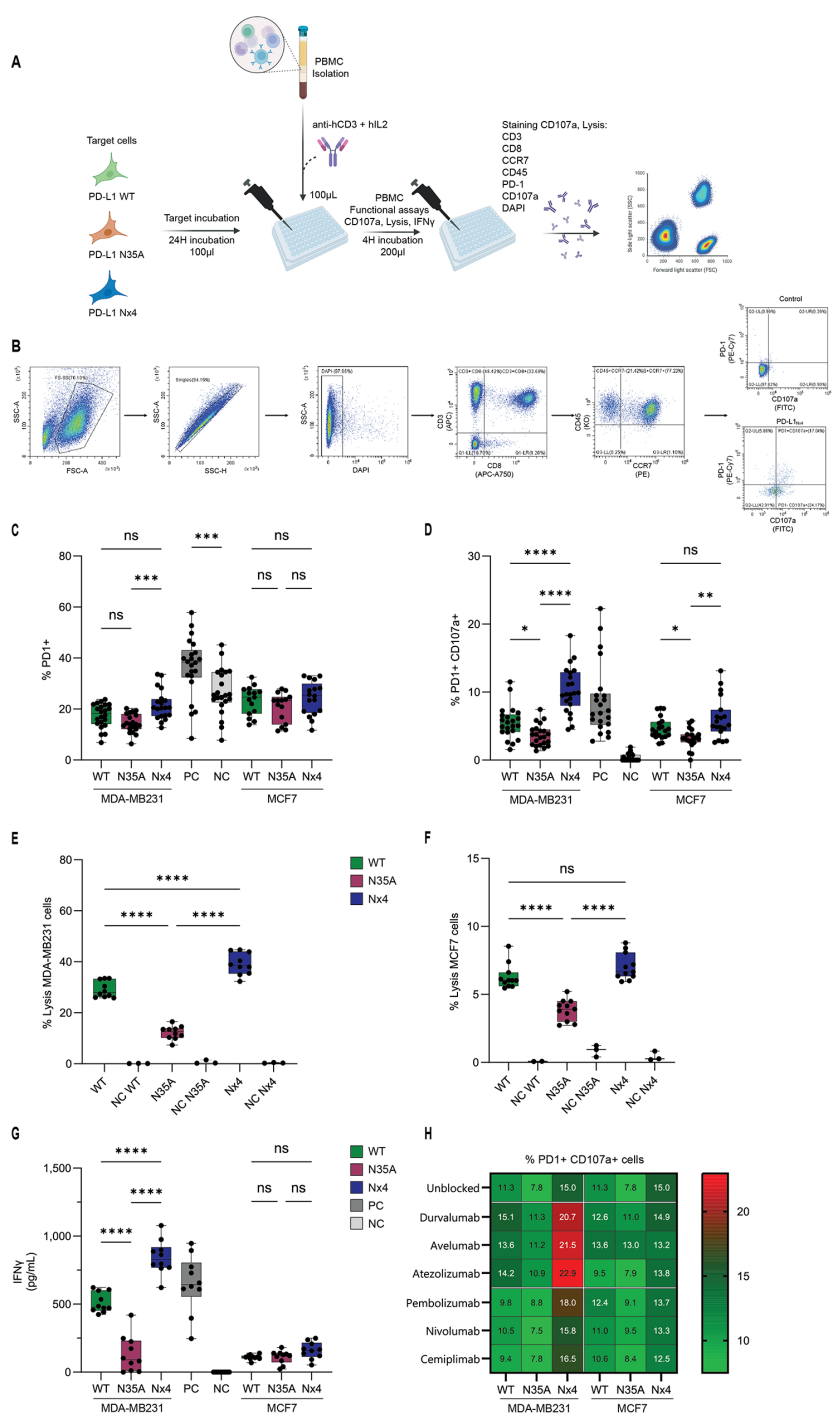
PD-L1_N35A_ suppresses the ability of anti-PD-L1 and anti-PD-1 ICBs to block the interactions of PD-L1 with PD-1 in CD8+ T_CM_ Cells, whereas ablation of N-glycosylation enhances ICB efficacy. **(A)** Schematic representation of the experimental procedure, determining the impact of PD-L1 N-glycosylation on T-cell activation and on the ability of anti-PD-L1 and anti-PD-1 ICBs to block PD-L1/PD-1 interactions. **(B)** Flow cytometry gating strategy. Representative flow cytometry plots of PBMCs, showing the gating strategy for identifying CD3+ CD8+ (CD45+ CCR7+) T_CM_ PD-1+ cells and assessing their CD107a expression. **(C)** PD-1 expression on CD8+ T_CM_ cells. Box plots depict the percentage of PD-1+ cells within the T_CM_ population after co-culture with MDA-MB231 cells expressing WT, N35A or Nx4 PD-L1. Each box represents data from nine donors with two data points per donor. Data are presented as median with interquartile range; whiskers extend to the minimum and maximum values. Statistical analysis was performed using Welch’s ANOVA. **(D)** Activation of CD8+ PD-1+ T_CM_ cells. Box plots demonstrate the percentage of CD107a+ cells within the CD8+ PD-1+ T_CM_ cells population following co-culture with WT-, N35A-, or Nx4-PD-L1 expressing cells. Each box represents data from nine donors with two data points per donor. Data are presented as median with interquartile range; whiskers extend to the minimum and maximum values. Statistical analysis was performed using Welch’s ANOVA. **(E) and (F)** Boxplots depicting the percent of lysis of target cells with and without (NC) PBMCs for (E) MDA-MB231 cells and (F) MCF7 cells. Each box represents data from five donors with two data points per donor. **(G)** Boxplots depicting the percent of IFNγ secreted by PBMCs in response to interaction with cancer cells. Each box represents data from nine donors with two data points per donor. **(H)** Heatmap depicting the percentage of CD107a+ cells within the CD8+ PD-1+ T_CM_ cell population in response to co-culture with WT, N35A or Nx4 PD-L1-expressing cells in the presence of anti-PD-L1 antibodies and anti-PD-1 antibodies. Each box represents data from five donors with two data points per donor. *p < 0.05, **p < 0.01, ***p < 0.001, ****p < 0.0001. NS = Not significant

We then sought to validate our findings in a clinically-relevant setting by examining the ability of MDA-MB231 and MCF7 target cells expressing PD-L1_WT_ and the PD-L1_N35A_ and PD-L1_Nx4_ mutations to influence the cytotoxic activities of CD8+ T-cells derived from peripheral blood mononuclear cells (PBMCs) of healthy donors. To that end, we co-cultured target tumor cells with naïve PBMCs co-activated by anti-hCD3 and rhIL-2. Then we measured PBMC functionality employing CD107a degranulation marker, IFNγ secretion and lysis of target cells (Scheme, Fig. 6A). Notably, in light of the potential roles of sPD-L1 in regulating the interactions of PD-L1 with PD-1 (Fig. 4), the cancer cells were grown in culture for 24 h prior to the 4-hour co-culturing with PMBCs to allow the accumulation of sPD-L1 in cell supernatant.

Using the aforementioned procedure, we examined PBMCs from nine different donors in two independent experimental repeats to ensure consistency. Following a 4-hour incubation with cancer cells, we stained the PBMCs to identify CD45+ CD3+ CD8+ CCR7+ T-cells (CD8+ T_CM_) and measured their expression of PD-1 and CD107a (Scheme, Fig. 6B).

First, we sought to understand how different PD-L1 variants affected PD-1 expression on CD8+ T_CM_ cells. Since PD-1 is an activation marker for T-cells [41], we measured its expression levels to determine the extent to which tumor cells inhibit T-cell activation. As expected, positive control cells that were activated by anti-hCD3 had a higher percentage of PD-1-expressing cells (36.5% ± 11.8%) compared to non-activated negative control cells (26.7% ± 9.0%, one-way ANOVA, *p* < 0.001). Additional exposure of anti-hCD3-activated T-cells to PD-L1 positive cancer cells should reduce membrane PD-1 expression due to internalization/release processes [42–44]. Indeed, incubation of anti-hCD3 activated CD8+ T_CM_ cells with PD-L1 positive tumor cells reduced membrane-PD-1 expression (Fig. 6C). For MDA-MB231 cells, incubation with PD-L1_Nx4_ cells showed the least reduction and PD-1-expressing T-cells were significantly higher (21.6% ± 5.8%) compared to CD8+ T_CM_ cells incubated with PD-L1_WT_ (17.4% ± 4.7 %, one-way ANOVA *p* < 0.05) or PD-L1_N35A_ cells (14.5% ± 3.6%, one-way ANOVA *p* < 0.0001). This could be attributed to the reduced interaction between the PD-L1_Nx4_ and PD-1, mediating less internalization/release of T-cell membrane PD-1. T-cells incubated with PD-L1_WT_ showed no difference in PD-1 expression levels compared to PD-L1_N35A_-expressing cells (Fig. 6C and Supplementary Fig. 6A). As for MCF7 cells, although T-cells incubated with PD-L1_Nx4_ expressing cells had higher levels of PD-1 (24.2% ± 6.6%) than those incubated with PD-L1_WT_ (23.3% ± 5.6%) or PD-L1_N35A_ (20.6% ± 5.8%), no statistical differences were found.

T-cell activation assays can provide additional evidence of the effect of various PD-L1 variants on T-cell activation by using CD107a expression as a marker for degranulation and thus for T-cell cytotoxicity (Fig. 6D and Supplementary Fig. 6B). Analyses of CD8+ PD-1+ CD107a+ T_CM_ cells showed that negative control cells that were not activated by anti-hCD3 or co-cultured with cancer cells had minor levels of CD107a-expressing cells (0.3% ± 0.54%) compared to positive control cells that were activated by anti-hCD3, which had significantly higher levels of cells expressing CD107a (8.7% ± 5.2%). Analysis of CD8+ PD-1+ CD107a+ T_CM_ cells from activated PBMC-cancer cell co-cultures showed that cells expressing PD-L1_WT_ resulted in lower levels of CD107a+ T-cells (5.5% ± 2.4%) compared to positive control T-cells activated without tumor cell exposure (8.7% ± 5.2%). While both MDA-MB231 and MCF7 cells exhibited similar patterns between mutants, MCF7 cells had lower capacity to induce T-cell degranulation, as indicated by the lower percentages of PD-1+ CD107a+ cells. In both cell lines, cells expressing the PD-L1_N35A_ variant had a lowest ability to activate T-cells (3.7% ± 1.5% for MDA-MB231 and 3.1% ± 1.4% for MCF7); however, the PD-L1_Nx4_ expressing cells had the highest ability to induce cytotoxic potential in CD8+ T_CM_ (10.3% ± 3.4% for MDA-MB231 and 6.1% ± 3.0% for MCF7). Again, reduced interaction between PD-L1_Nx4_ to PD-1 could explain the higher membrane-associated CD107a observed for anti-hCD3-activated CD8+ T_CM_ co-incubated with the PD-L1_Nx4_ target cells.

To further characterize how PD-L1 mutants affect PBMC activation and cytotoxicity, we conducted a killing assay in which each PD-L1 mutant cell line was incubated with PBMCs from five different donors. We evaluated cell lysis and measured interferon γ (IFNγ) secretion in the supernatants via ELISA. For lysis, similar to the degranulation assay, MDA-MB231 cells showed greater capacity to induce lysis (Fig. 6E) than MCF7 cells (Fig. 6F). The pattern of killing remained consistent across individual mutants in both cell lines. Cells expressing PD-L1_N35A_ demonstrated the least lysis (12.2% ± 2.1% for MDA-MB231 and 3.8% ± 0.7% for MCF7), while PD-L1_Nx4_ expressing cells induced the highest killing (39.1% ± 4.2% for MDA-MB231 and 7.0% ± 0.9% for MCF7). This pattern was mirrored in IFNγ secretion (Fig. 6G). While PBMCs incubated with MDA-MB231 cells secreted higher levels of IFNγ than those with MCF7 cells, the relative differences between mutants remained consistent for MDA-MB231, but no differences were found for MCF7 cells, reflecting its lower level to activate PBMCs in our assays.

We then investigated how anti-PD-L1 or anti-PD-1 antibodies affected the degranulation activity of CD8+ T_CM_, using pooled samples from five donors to ensure reliable results (Fig. 6H and Supplementary Fig. 6C and D). As demonstrated in Fig. 6D, also in this test negative control cells demonstrated lower levels of CD8+ PD-1+ CD107a+ cells, compared to positive control cells (Supplementary Fig. C1, C2, D1, D2). When PD-L1_WT_ MDA-MB231 cells were added, all ICBs directed to PD-L1 blocked PD-L1/PD-1 and have led to elevated levels of such CD8+ cells. Importantly, the extent of CD8+ PD-1+ CD107a+ cells was further elevated when PD-L1_Nx4_ cells were used. These patterns remained similar, regardless of the antibody at use, indicating that anti-PD-L1 antibodies enhance the cytotoxic potential of CD8+T_CM_, especially towards PD-L1_Nx4_ mutant. In parallel, in the presence of PD-L1_N35A_-expressing cells, the levels of CD8+ T_CM_ were reduced compared to PD-L1_WT_-expressing cells. Similar but less pronounced effects were observed with PD-1-targeting ICBs, where CD8+ PD-1+ CD107a+ cell generation was improved with PD-L1_Nx4_ and cells compared to PD-L1_WT_ cells. The stronger effect of anti-PD-L1 antibodies compared to anti-PD-1 antibodies parallels our findings in Figs. 2 and 3. Together, these results align with our IcAR-PD-1 reporter system findings, demonstrating that complete PD-L1 N-glycosylation reduces ICB efficacy, while N35 glycosylation enhances ICB blocking activity. the blocking activities of the ICBs.

These findings suggest that N-glycosylation at all four PD-L1 sites impairs ICBs’ ability to block PD-L1/PD-1 interactions, as previously demonstrated with the IcAR-PD-1 system in which mutation in all 4 sites (Nx4) enhanced ICBs’ ability to block (Figs. 2 and 3).

## Discussion

This study offers new perspectives into the biological relevance of PD-L1 N-glycosylation, using a functional bioavailability assay with immune-checkpoint reporter overexpressing human PD-1 (IcAR-PD-1) [33, 34] and further validation using primary T-cell degranulation and cytotoxicity assays. Using these research approaches, we provide insights to two questions: (1) Whether PD-L1 N-glycosylation plays a role in determining the efficacy of PD-L1 binding to PD-1? (2) Whether PD-L1 N-glycosylation impacts the blocking efficacy of ICBs targeting PD-L1 or PD-1?

Our study refines previous information suggesting that PD-L1 N-glycosylation is required for efficient PD-1 binding, using soluble PD-1 and antibodies recognizing some of the N-glycosylation sites of PD-L1 [45]. Specifically, we have provided data on the roles of each N-glycosylation site in the process, when both PD-L1 and PD-1 are expressed as intact cell surface-attached molecules. Our data in both cell systems, of MDA-MB231 and MCF7 cells, indicate that none of the four N-glycosylation sites of PD-L1 is required, on its own, for efficient binding and activation of PD-1.

Moreover, in MDA-MB231 cells, the entire array of four N-glycosylation sites was dispensable in terms of PD-1 binding, as illustrated by the fact that the PD-L1_Nx4_ mutant has given rise to the same levels of mIL-2 production by the IcAR-PD-1 reporter system as did the WT protein, despite the lower cell surface expression levels of PD-L1 on PD-L1_Nx4_ cells than PD-L1_WT_ cells. The reduced expression of PD-L1_Nx4_ at the cell membrane is possibly connected to its reduced N-glycosylation: Previous reports indicated that PD-L1 N-glycosylation protects the protein from degradation [18, 46, 47], and that increased PD-L1 stability is achieved by TMUB1; specifically, TMUB1 was found to enhance PD-L1 N-glycosylation and to inhibit PD-L1 polyubiquitination at the K281 residue. Moreover, removal of PD-L1 N-glycosylation was suggested to cause ER retention [21, 48], and to suppress the formation of PD-L1 membrane homodimers [49], giving rise to reduced membrane expression [50]. Together, these findings suggest that that the lower expression of PD-L1_Nx4_ at the cell surface stems from alterations in its intracellular processing.

However, as mentioned above, despite lower expression at individual time points, sufficient PD-L1 proteins were present on the cell surface of PD-L1_Nx4_-expressing cells over the 24-hour period of the test to effectively engage PD-1. This observation may reflect a spatio-temporal mechanism for PD-L1 function that is involved in determining its binding efficacy to PD-1. Indeed, in our study we show high total-cellular-to-membrane ratio of PD-L1 in PD-L1_Nx4_-expressing cells, suggesting a rapid turnover of membrane-bound PD-L1. In accordance, abolishing the turnover through fixing PD-L1_Nx4_ cells significantly reduced its functionality.

A different role was revealed for the entire array of N-glycosylation site in MCF7 cells, where the PD-L1_Nx4_ mutant did not bind efficiently to PD-1, indicating that in this case the mechanisms regulating PD-L1 binding to PD-1 are different than in MDA-MB231 cells. This may be due to differences between the two cell types in the type of glycoforms expressed by PD-L1 in each of them. This possibility is supported by a recent study demonstrating high heterogeneity in the types of N-glycans carried by cell surface-expressed PD-L1 in different cell types [23] suggesting that the specific composition of PD-L1 N-glycans in MDA-MB231 and MCF7 cells regulates differently the interactions between PD-L1 and PD-1 in each cell type. In parallel, the differences between the two cell types may be connected to the different biological-immunological nature of MDA-MB231 and MCF7 cells, and may provide insights that are relevant to TNBC being “hot” tumors that may raise better anti-immune activities than the “cold” ER+ breast tumors.

The entire N-glycan array of PD-L1 was also found in our study to regulate the blocking efficacy of PD-L1/PD-1 by ICBs targeting PD-L1. Specifically, in both MDA-MB231 and MCF7 cells, the blocking activity of ICBs targeting PD-L1 was improved when PD-L1 was stripped off its N-glycans by using the PD-L1_Nx4_ mutant. These findings may reflect a steric interference caused by PD-L1 glycoforms, inhibiting the binding of ICBs to PD-L1. This possibility is in line with published observations, indicating that deglycosylation of PD-L1 enables the recognition of PD-L1 by antibodies in patient biopsies that were considered PD-L1-negative prior to deglycosylation [51].

In parallel, a striking finding was obtained in our study, demonstrating that in both MDA-MB231 and MCF7 cells, the coverage of PD-L1 by N-glycans at all four sites interfered with the blocking potential of ICBs targeting PD-1. Here, it is interesting to note that Cemiplimab, one of the clinical PD-1-targeting ICBs used in our study, binds directly to fucose at N58 of PD-1 in CHO cells [52]. While direct binding to fucose was not found to be required for Nivolumab and Pembrolizumab binding to PD-1, it is possible that the N-glycan composition of PD-1 in our system does lead to the binding of all PD-1-targeting ICBs to N-glycans. In such case, the full N-glycosylation of PD-L1 may sterically interfere with the binding of the ICBs to the N-glycans of PD-1. Another possibility is that the low expression of PD-L1 at any given time point and its minimal release gives rise to reduced PD-L1/PD-1 interactions per time point. Under these conditions, even low antibody concentrations can effectively block these interactions, as indeed demonstrated in our findings.

Our data also reveal that the N35 glycosylation site of PD-L1 plays a crucial role in regulating the blocking efficacy of ICBs directed to PD-L1 and to PD-1. We have demonstrated that PD-L1_N35A_ exhibited increased levels of sPD-L1 that potently enhanced PD-1 activation. In the presence of ICBs, such high levels of sPD-L1 may lead to retained and continuous activation of PD-1 despite the presence of the IBCs, thus interfering with their blocking efficacy. Supporting this mechanism is the observation that under fixed-cell conditions where no soluble forms of PD-L1 were available, PD-L1_N35A_ displayed lower ability to interfere with anti-PD-1 blockade, particularly with Cemiplimab. Along these lines, others have shown that sPD-L1 reduces the function of CD8+ T_CM_ cells [53, 54]. Accordingly, recent reports suggested that soluble forms of PD-L1 can serve as a prognostic marker to the efficacy of anti PD-L1 and anti PD-1 therapy.

Activities of the soluble form of PD-L1 may be detected due to the presence of the protein in exosomes, where it carries functional orientation, enabling it to binding to PD-1 and induce immune suppression in cancer [55]. Indeed, the WT and mutant forms of PD-L1 were found to be expressed in EVs produced by MDA-MB231 and MCF7 cells, but at lower levels than the soluble form of the protein, sPD-L1. Of importance was the fact that sPD-L1 generated by the N35 PD-L1-mutated cells was highly effective in activating PD-1, as observed using the IcAR-PD-1 cellular system. sPD-L1 is known to be obtained by cleavage of the membrane protein by ADMA17 and ADAM10 [56]. The exacerbated levels of sPD-L1 demonstrated for the PD-L1_N35A_ mutant may reflect a conformation change that facilitates the accessibility of these enzymes to the cleavage point of the protein. Supporting this possibility are observations on glycosylation-induced conformational changes in proteins [57–59] and findings demonstrating that protein domains having different conformations affect the blocking potential of anti PD-1 antibodies [60]. Such conformation changes can also affect the interactions between PD-L1 and PD-1 upon modified N-glycosylation, particularly when the N35 residue of PD-L1 is involved. This residue is located at the IgV-like domain of PD-L1, where conformation flexibility was observed following the binding of the two proteins to each other.

Our ex vivo T-cell degranulation and cytotoxicity assays using freshly isolated anti-hCD3 activated PBMCs from healthy donors provided crucial validation to the findings obtained with the IcAR-PD-1 cell system, in a physiologically relevant context. This assay revealed distinct patterns of T-cell modulation by different PD-L1 variants, aligning with our IcAR-PD-1 functional bioavailability assay: T-cell degranulation was substantially increased in the presence of ICBs targeting PD-L1 and PD-1 when the T-cells were co-cultured with tumor cells expressing PD-L1_Nx4_, compared to T-cell co-cultures with PD-L1_WT_-expressing cells; these findings indicate that PD-L1 N-glycosylation interferes with the ability of ICBs to block PD-L1/PD-1 interactions, and thus to reactivate cytotoxic T-cells. Also agreeing with our results with the IcAR reporter system are the findings obtained with the T-cell degranulation function with regards to the PD-L1_N35A_ mutant. Anti-CD3 activated CD8+ T_CM_ cells incubated with PD-L1_N35A_-expressing cancer cells showed the lowest degranulation and lysis in the presence of ICBs to PD-L1 or PD-1, significantly lower than PD-L1_WT_, indicating higher inhibition. This effect is likely enhanced by the presence of sPD-L1, which has been shown to suppress CD8+ T_CM_ cell activation and proliferation [53, 61]. The reduced lysis observed for PD-L1_N35A_ mutants highlights the added value of the model of ex vivo anti-CD3 activated T cells as compared to the in vitro IcAR-PD-1 model, that did not identify the ability of PD-L1_N35A_ mutant to activate PD-1 differently than the WT protein. These findings suggest that a two step model could be used for clinical needs, where the IcAR-PD-1 system can provide initial screening, and the most appropriate PD-L1 constructs are then further investigated by using the ex vivo T-cell system, which is much more laborious.

Moreover, we conducted a killing assay in which each PD-L1 mutant cell line was incubated with PBMCs from five different donors. The pattern of killing remained consistent across individual mutants in both MDA-MB231 and MCF7 cell lines. Cells expressing PD-L1_N35A_ demonstrated the least lysis, while PD-L1_Nx4_ expressing cells induced the highest killing. This pattern was mirrored in IFNγ secretion, with higher levels observed in PBMCs incubated with MDA-MB231 cells compared to MCF7 cells, possibly reflecting the intrinsic immunogenicity of the cell lines. MDA-MB231 cells induced greater immune responses, which correlates with their more immune-responsive nature [62].

The different effects of PD-L1 N-glycosylation status on the ability of ICBs to inhibit the interactions of PD-L1 with PD-1 expressed by CD8+ T_CM_ cells demonstrate the immediate functional consequences of PD-L1 glycosylation alterations. They indicate that the glycosylation status of PD-L1 directly impacts its immunosuppressive function in a more complex cellular environment. The consistency between our molecular/cellular assays and this ex vivo T-cell model strengthens the physiological relevance of our observations and underscores the potential clinical implications of PD-L1 glycosylation variants.

Our findings offer potential explanations for the inconsistent associations between PD-L1 staining and patient response to ICBs. The PD-L1_Nx4_ mutant, which showed low membrane staining but retained functionality, could represent cases where patients with low PD-L1 staining unexpectedly respond to ICB therapy [25]. Conversely, the PD-L1_N35A_ mutant, with similar staining to PD-L1_WT_, but increased release and blockade resistance, might explain cases where patients with high PD-L1 staining fail to respond [63–65].

These observations underscore the need to extend the analyses on PD-L1 N-glycosylation from the study of cell lines to more comprehensive assessments of PD-L1 N-glycosylation status in patient tumors, possibly enabling improved patient stratification and treatment selection for ICB therapies. Previous studies - demonstrating that PD-L1 N-glycosylation interferes with detection of PD-L1 in patient biopsies - emphasized the need to determine the N-glycosylation status of PD-L1 in patient biopsies for diagnostic needs. The findings of our study, demonstrating that N-glycosylation of PD-L1 at all four sites interferes with the blocking activities of ICBs directed to PD-L1 or PD-1, suggest that patients whose tumors have low N-glycosylation levels would potentially respond better to such ICBs than patients whose cancer express high N-glycosylation levels. These observations emphasize the need to determine the status of PD-L1 N-glycosylation, for example by using antibodies recognizing the N-glycosylated form of PD-L1. Such antibodies have been characterized in previous studies, demonstrating that the antibodies STM004 and STM108 recognize the glycan moieties of PD-L1, at positions N35 and N192/N200, respectively. The use of such antibodies, or others that recognize different N-glycosylated forms of PD-L1 in patient biopsies, should be taken into account when ICB therapies are considered in the treatment of each specific patient.

While our study provides valuable insights into PD-L1 N-glycosylation and immune checkpoint interactions, several limitations should be noted. The variability of N-glycans attached to PD-L1 in different cell types suggests that tumor cell heterogeneity may hinder effective and accurate determination of the levels of types and levels of N-glycosylation in patient tumors. In addition, while our ex vivo degranulation and cytotoxicity assays provide crucial validation, it represents a simplified model of the tumor microenvironment [66]. Our focus on specific glycosylation mutants may not capture the full spectrum of post-translational modifications affecting PD-L1 function [67]. Additionally, while we observed significant effects of PD-L1 glycosylation on antibody blockade in vitro, the long-term clinical implications remain to be determined. Future studies should address these limitations by using more complex in vivo systems and conducting longitudinal studies in patients receiving immune checkpoint inhibitors.

## Conclusion

In conclusion, this study reveals the significant role of PD-L1 glycosylation in modulating its function and interaction with PD-1 and with ICBs targeting the two partners of the PD-L1/PD-1 axis. Our findings emphasize the complexity of immune checkpoint regulation and suggest that variations in PD-L1 N-glycosylation can influence T-cell responses. This understanding opens new avenues for enhancing cancer immunotherapy strategies by targeting the mechanisms underlying PD-L1/PD-1 interactions, potentially leading to more effective treatments for patients.

## Electronic supplementary material

Below is the link to the electronic supplementary material.

Supplementary Material 1

## Data Availability

No datasets were generated or analysed during the current study.

## Abbreviations

ICB: Immune Checkpoint Blockade
PD-1: Programmed Cell Death Protein-1
PD-L1: Programmed Death-Ligand 1
sPD-L1: soluble Programmed Death-Ligand 1
WT: Wild Type
mIL2: Murine Interleukin-2
T_CM_: T central memory
PBMC: Peripheral Blood Mononuclear Cell
IHC: Immunohistochemistry
IcAR-PD-1: Immune Checkpoint Artificial Reporter overexpressing PD-1
