# Supplementary material for: N-glycosylation of PD-L1 modulates the efficacy of immune checkpoint blockades targeting PD-L1 and PD-1
Source: Mol Cancer. 2025 May 10;24:140. doi: 10.1186/s12943-025-02330-w (PMC12065222; doi:10.1186/s12943-025-02330-w)
Supplement: Supplementary file 1 — Supplementary Material 1 [file 12943_2025_2330_MOESM1_ESM.docx]

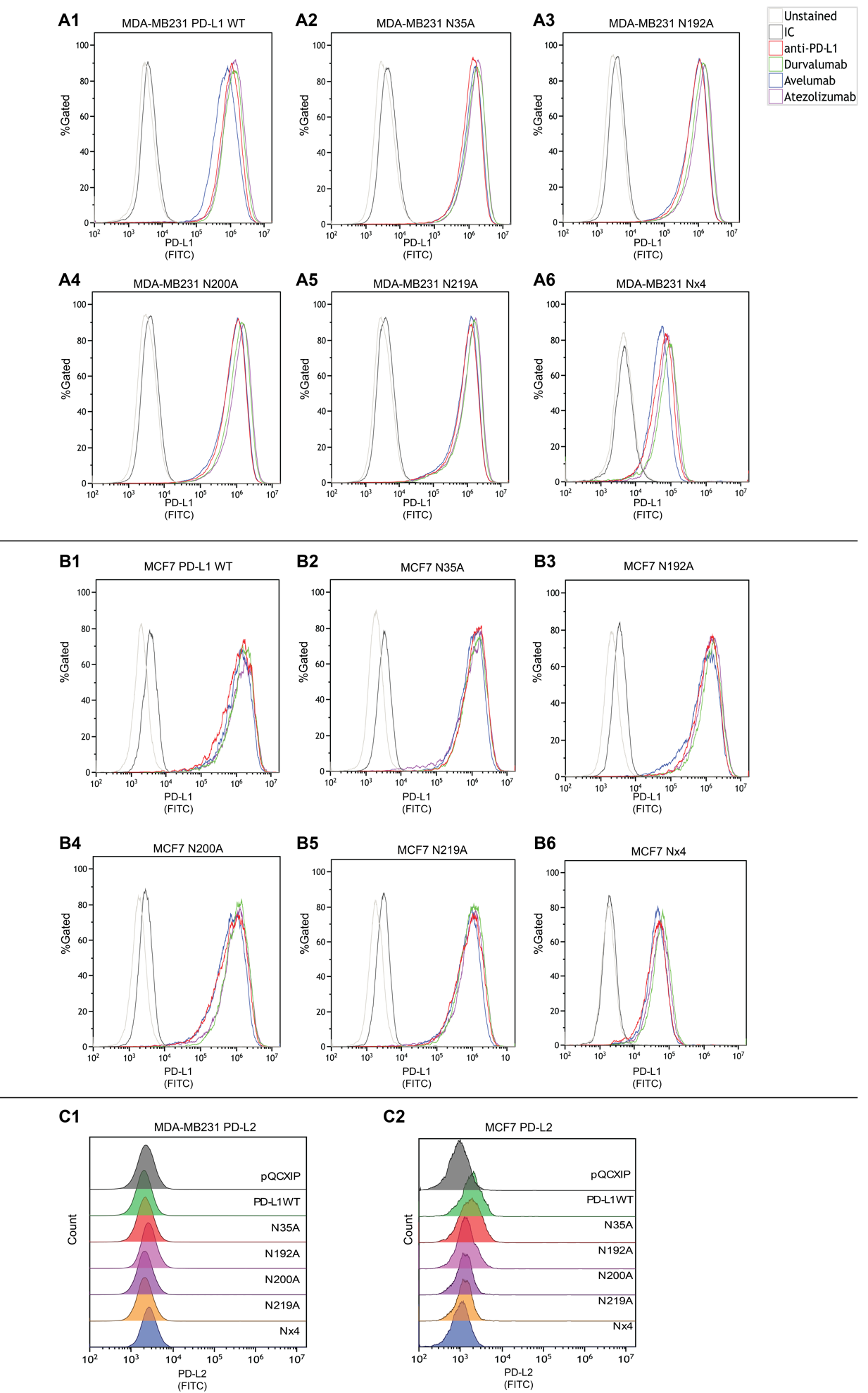


**Supplementary Figure 1**

**PD-L1_WT_ and PD-L1 N-glycosylation mutants are well detected by clinical antibodies to PD-L1 in MDA-MB231 and MCF7 cells, with minimal detection (if any) of PD-L2**

**A.** and **B.** Histograms depicting PD-L1 expression of PD-L1_WT_ and PD-L1 N-glycosylation variants. Isotype controls (IC) were used in parallel to commercial anti-PD-L1 antibodies (termed anti-PD-L1), and clinical anti-PD-L1 antibodies: Atezolizumab, Avelumab and Durvalumab at 2 μg/mL for MDA-MB231 cells (**A1-A6**) and MCF7 cells (**B1-B6**) variants. **C.** Histograms demonstrating PD-L2 expression by MDA-MB231 cells (**C**) and MCF7 cells (**D**) expressing PD-L1_WT_ or different PD-L1 N-glycosylation mutants, using commercial anti-PD-L2 antibody at 2 μg/mL. Histograms shown are representative of three independent experiments performed in triplicate.

**
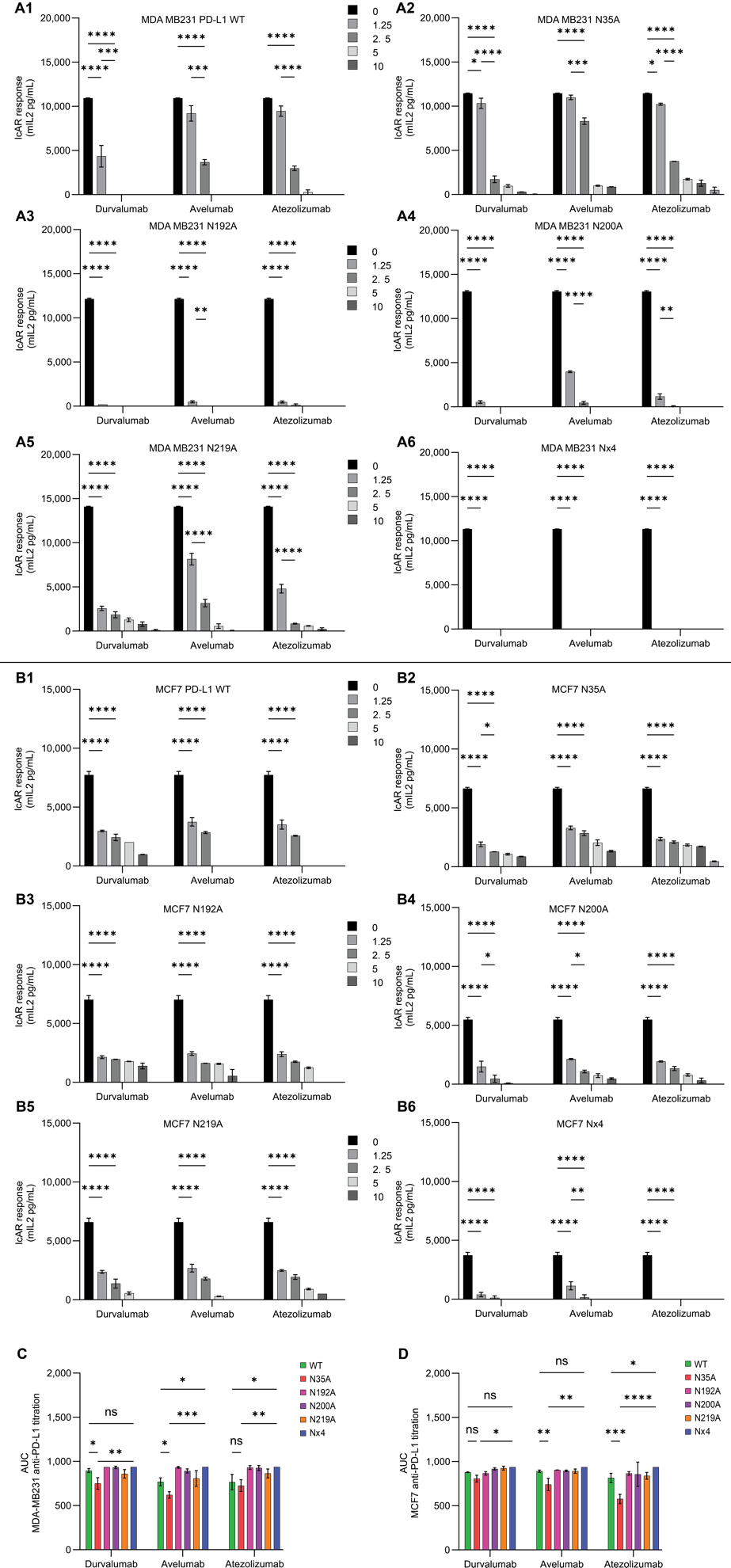
**

**Supplementary Figure 2**

**Complementary data to Figure 2, demonstrating the ability of anti-PD-L1 ICBs to block PD-L1/PD-1 interactions in cancer cells expressing PD-L1_WT_ or PD-L1 N-glycosylation mutants**

Bar charts demonstrating raw mIL2 levels produced by IcAR-PD-1 cells co-cultured with MDA-MB231 cells (**A1-A6)** and MCF7 cells **(B1-B6)** expressing PD-L1_WT_ or N-glycosylation mutants, in the presence of anti-PD-L1 clinical antibodies (Atezolizumab, Avelumab and Durvalumab) at concentrations ranging from
0 μg/mL to 20 μg/mL. Bars represent mean ± SEM from three triplicate biological repeats. **C. and D.** AUC comparison of blocking assay with anti-PD-L1 antibodies of MDA-MB231 (C) and MCF7 (D) PD-L1 mutants. Bar graphs comparing area under the curve (AUC) values for PD-L1_WT_, N35A mutant, and Nx4 mutant with Durvalumab, Avelumab, and Atezolizumab. Y-axis represents AUC values. Bars show mean values with SEM. One-way ANOVA with Dunnett's multiple comparisons test assessed differences between WT and mutant PD-L1 for each antibody.

*p < 0.05, **p < 0.01, ***p < 0.001, ****p < 0.0001. NS = Not significant.

**
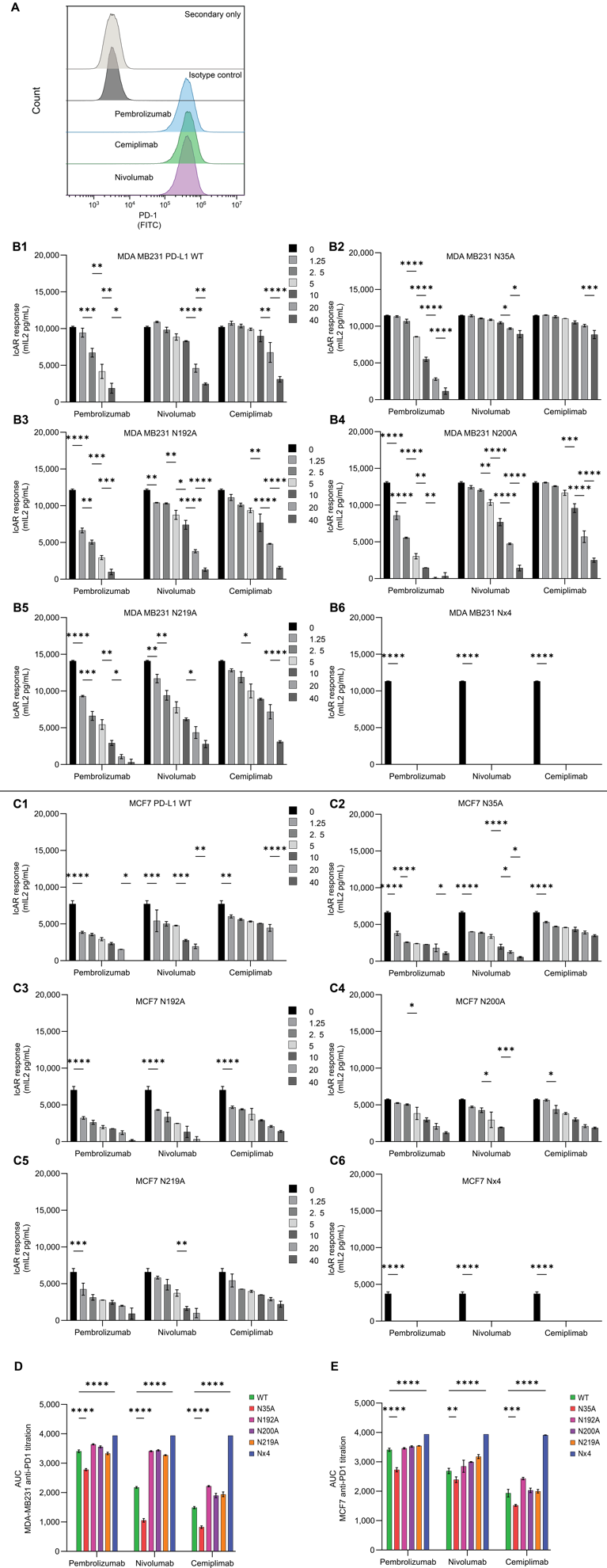
Supplementary Figure 3**

**Complementary data to Figure 3, demonstrating the ability of anti-PD-1 ICBs to block PD-L1/PD-1 interactions in cancer cells expressing PD-L1_WT_ or PD-L1 N-glycosylation mutants**

**A.** Flow cytometry analysis demonstrating the detection of PD-1 by clinical anti-PD-1 antibodies, in IcAR-PD-1 cells. **B.** and **C**. Bar charts demonstrating raw mIL2 levels produced by IcAR-PD-1 cells co-cultured with MDA-MB231 cells (**A1-A6)** and MCF7 cells **(B1-B6)** expressing WT PD-L1 or N-glycosylation mutants, in the presence of anti-PD-1 clinical antibodies (Pembrolizumab, Nivolumab and Cemiplimab) at concentrations ranging from 0 μg/mL to 40 μg/mL. Bars represent mean ± SEM from three triplicate biological repeats. **D. and E.** AUC comparison of blocking assay with anti-PD-1 antibodies of MDA-MB231 (D) and MCF7 (E) PD-L1 mutants. Bar graphs comparing area under the curve (AUC) values for PD-L1_WT_, N35A mutant, and Nx4 mutant with Pembrolizumab, Nivolumab, and Cemiplimab. Y-axis represents AUC values. Bars show mean values with SEM. One-way ANOVA with Dunnett's multiple comparisons test assessed differences between WT and mutant PD-L1 for each antibody.

*p < 0.05, **p < 0.01, ***p < 0.001, ****p < 0.0001. NS = Not significant.


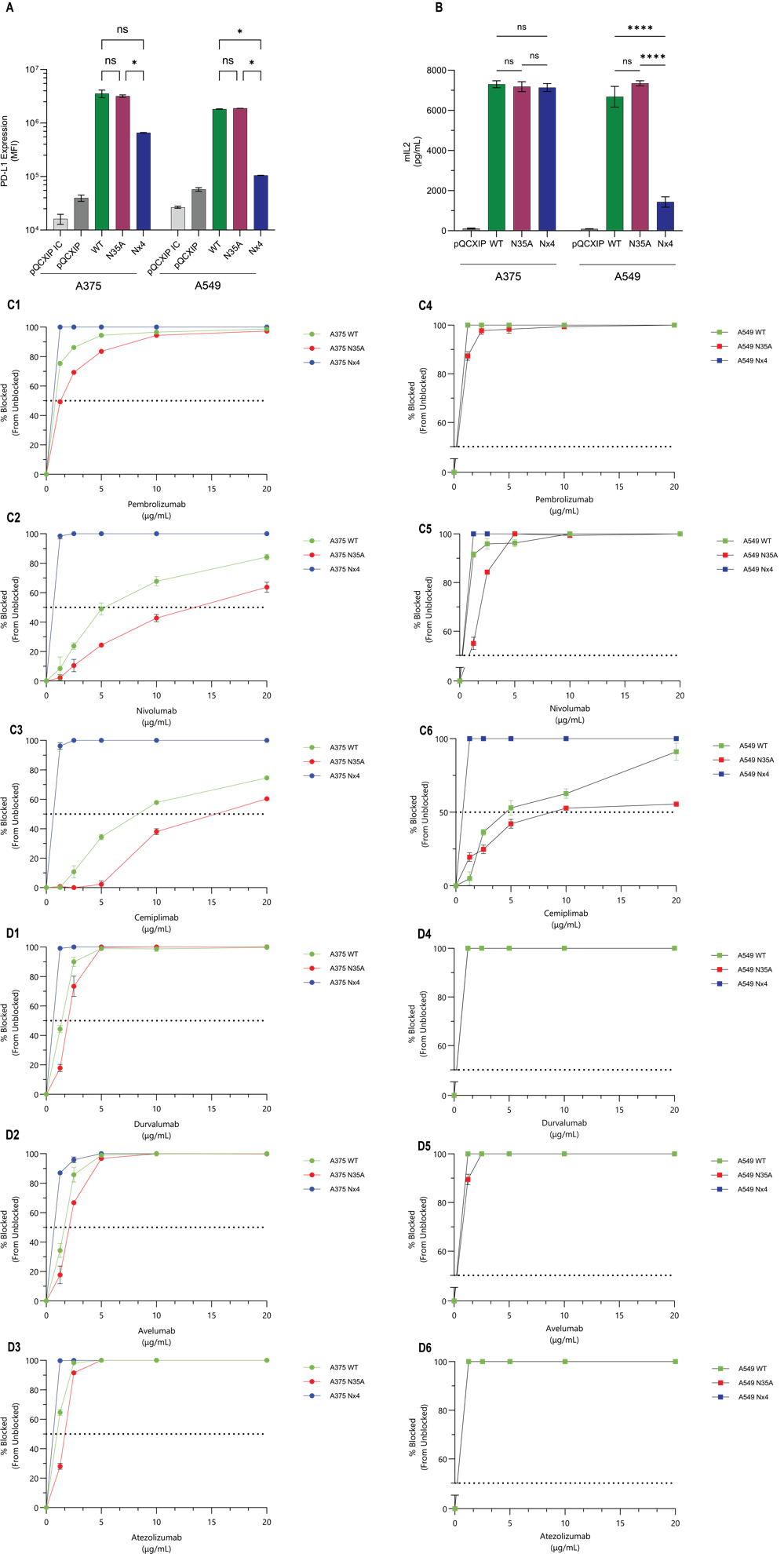
**Supplementary Figure 4**

**Validation of PD-L1 variant expression and ICB response in A375 and A549 cell lines**

**A.** Flow cytometry analysis demonstrating the detection of PD-L1 by commercial anti-PD-L1 antibody.  **B.** Bar chart depicting IcAR-PD-1 response to the vector only (pQCXIP), PD-L1_WT_, N35A and Nx4 PD-L1 variants is depicted by secretion of mIL2. Bars show mean values with SEM from triplicate biological repeats. One-way ANOVA with Dunnett's multiple comparisons test assessed differences between WT and mutant PD-L1 variants **C.** The capacity of anti-PD-1 antibodies in blocking the interactions of PD-L1 (WT and glycosylation variants) for A375 (left panel) and A549 (right panel). Graphs demonstrate the normalized blocking capacity of Pembrolizumab (C1 and C4), Nivolumab (C2 and C5), and Cemiplimab (C3a and C6). **D.** The capacity of anti-PD-L1 antibodies in blocking the interactions of PD-L1 (WT and glycosylation variants) for A375 (left panel) and A549 (right panel). Graphs demonstrate the normalized blocking capacity of Atezolizumab (D1 and D4), Avelumab (D2 and D5), and Durvalumab (D3 and D6) on each cell type. Antibody concentrations ranged from 0 μg/mL to 20 μg/mL. Data points represent mean ± SEM from triplicate biological repeats. Antibody concentrations ranged from 1.5 μg/mL to 20 μg/mL. Data points represent mean ± SEM from triplicate biological repeats.

 *p < 0.05, ****p < 0.0001. NS = Not significant.

In charts C4, D4-D6, data points are superimposed, representing overlapping values


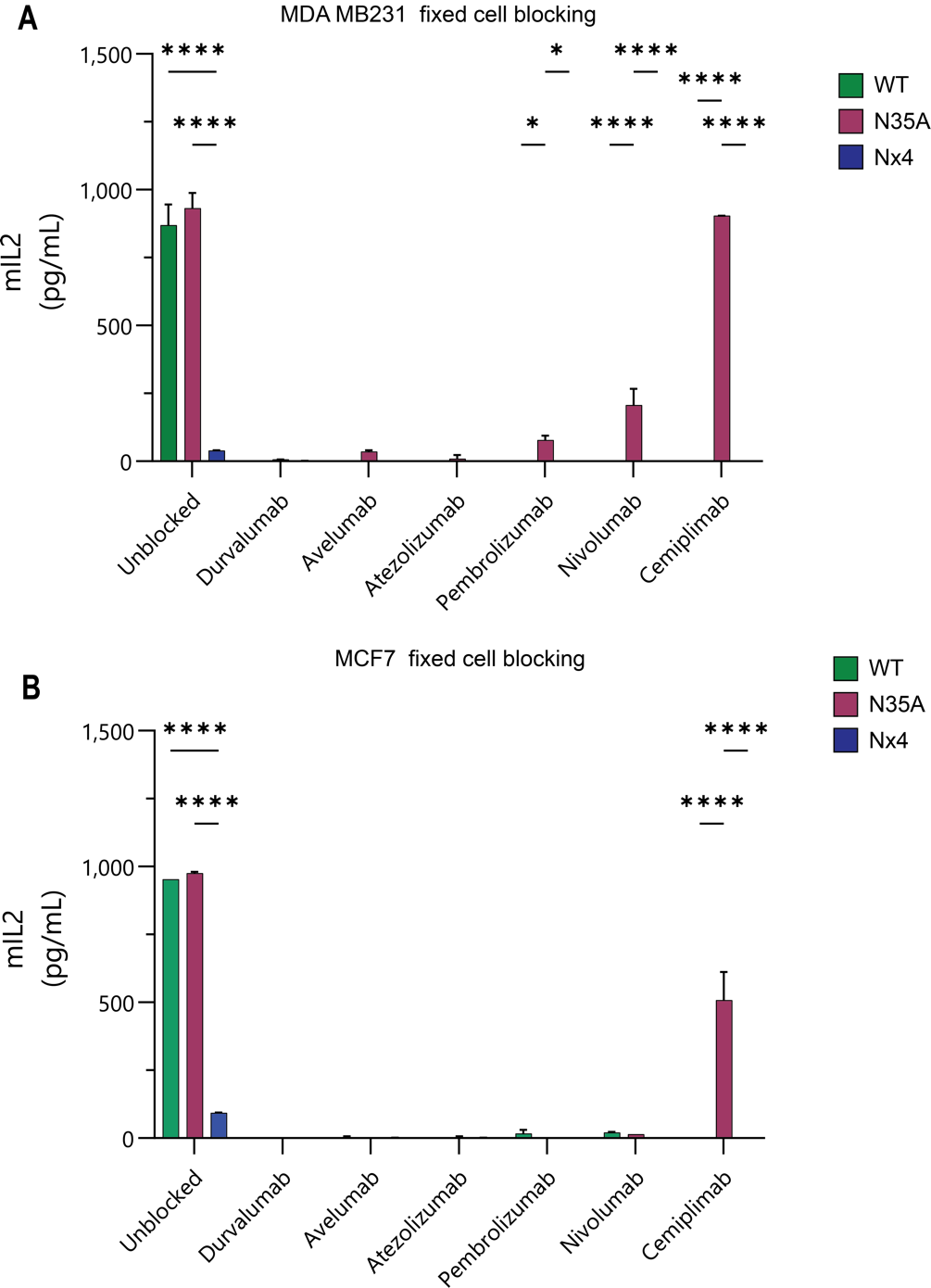


**Supplementary Figure 5.**

**A. and B.** Bar charts depicting raw mIL2 IcAR-PD-1 response to unblocked fixed target cells, and blocked cells with both anti-PD-L1 and anti-PD-1 antibodies, for both MDA-MB231 (**A**) and MCF7 (**B**) cells. Bars represent mean ± SEM from triplicate biological repeats.

 *p < 0.05, ****p < 0.0001.

**
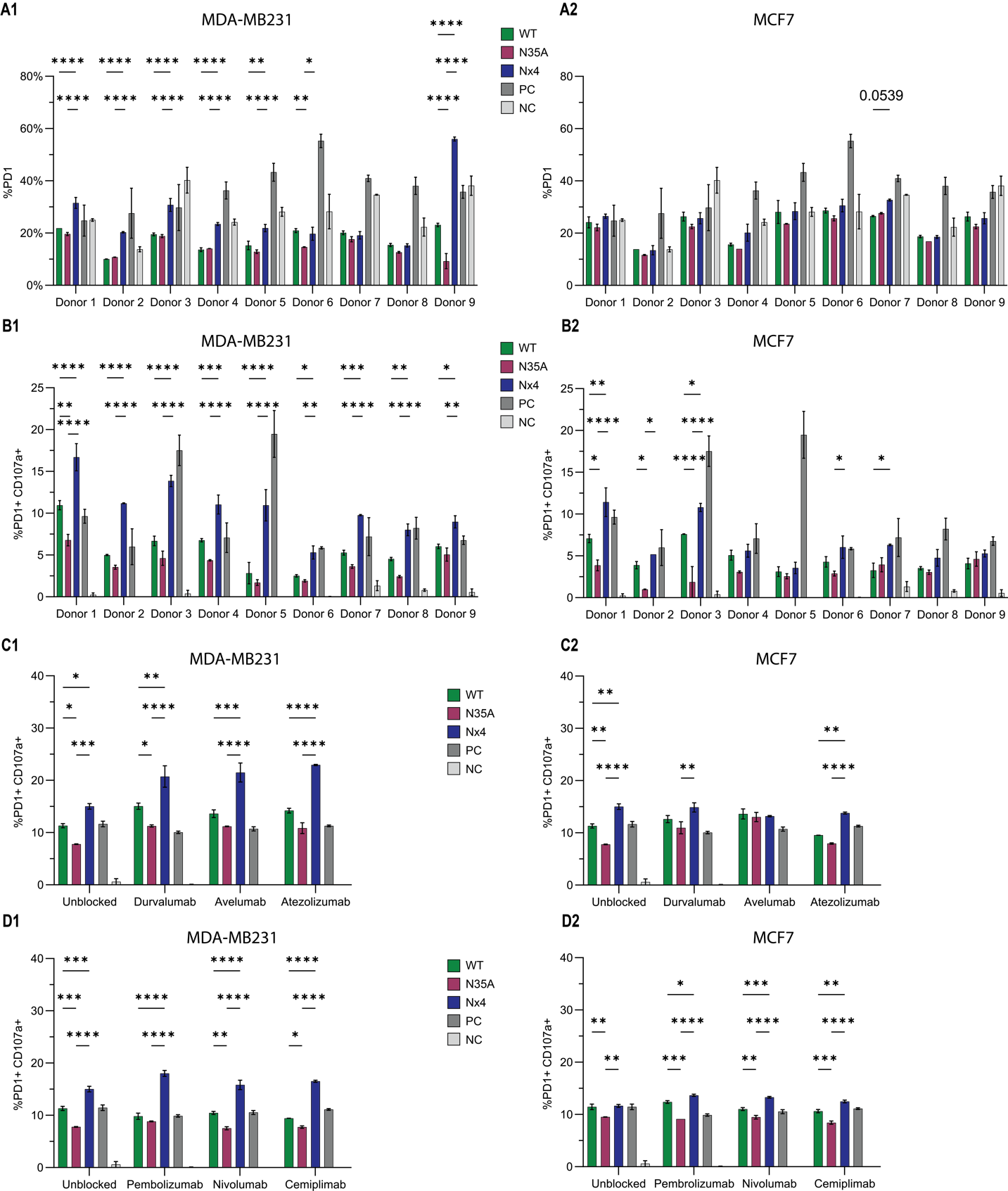
**

**Supplementary Figure 6**

**Complementary data to Figure 6, demonstrating the impact of different PD-L1 glycosylation mutants on the ability of anti-PD-L1 and anti-PD-1 ICBs to block the interactions of PD-L1 with PD-1 expressed by CD8^+^ T_CM_ cells**

**A.** Bar charts demonstrating the percent of PD-1-expressing CD8+ central memory T-cells (T_CM_) of individual donors, following co-culture with (A1) MDA-MB231 cells and (A2) MCF7 cells expressing PD-L1_WT_, N35A or Nx4 PD-L1 variants. NC and PC are as in Figure 6. **B.** Bar charts demonstrating the percent of activation (determined by CD107a expression) of CD8+ PD-1+ T_CM_ cells of individual donors incubated with (B1) MDA-MB231 cells and (B2) MCF7 cells. **C.** Bar charts demonstrating the effect of anti-PD-L1 ICB on the percent of activation (determined by CD107a expression) of CD8+ PD-1+ T_CM_ cells of individual donors incubated with (C1) MDA-MB231 cells and (C2) MCF7 cells. **D.** Bar charts demonstrating the effect of anti-PD-1 ICB on the percent of activation (determined by CD107a expression) of CD8+ PD-1+ T_CM_ cells of individual donors incubated with (D1) MDA-MB231 cells and (D2) MCF7 cells. Bars represent mean ± SEM from triplicate biological repeats. One-way ANOVA was performed to compare response across mutations within each donor.

 *p < 0.05, **p < 0.01, ***p < 0.001, ****p < 0.0001. NS = Not significant.
